# [2 + 2] cycloaddition and its photomechanical effects on 1D coordination polymers with reversible amide bonds and coordination site regulation†

**DOI:** 10.1039/d3sc06098e

**Published:** 2024-02-05

**Authors:** Lei Wang, Si-Bo Qiao, Yan-Ting Chen, Xun Ma, Wei-Ming Wei, Jun Zhang, Lin Du, Qi-Hua Zhao

**Affiliations:** a Key Laboratory of Medicinal Chemistry for Natural Resource, Ministry of Education and Yunnan Province, Yunnan Characteristic Plant Extraction Laboratory, School of Chemical Science and Technology, School of Pharmacy, Yunnan University 650500 People's Republic of China qhzhao@ynu.edu.cn lindu@ynu.edu.cn; b New Energy Photovoltaic Industry Research Center, Qinghai University Xining 810016 People's Republic of China

## Abstract

Photo-responsive materials can convert light energy into mechanical energy, with great application potential in biomedicine, flexible electronic devices, and bionic systems. We combined reversible amide bonds, coordination site regulation, and coordination polymer (CP) self-assembly to synthesize two 1D photo-responsive CPs. Obvious photomechanical behavior was observed under UV irradiation. By combining the CPs with PVA, the mechanical stresses were amplified and macroscopic driving behavior was realized. In addition, two cyclobutane amide derivatives and a pair of cyclobutane carboxyl isomers were isolated through coordination bond destruction and amide bond hydrolysis. Therefore, photo-actuators and supramolecular synthesis in smart materials may serve as important clues.

## Abstract 2

Photo-responsive materials can convert light energy into mechanical energy, with great application potential in biomedicine, flexible electronic devices, and bionic systems.

## Graphical abstract

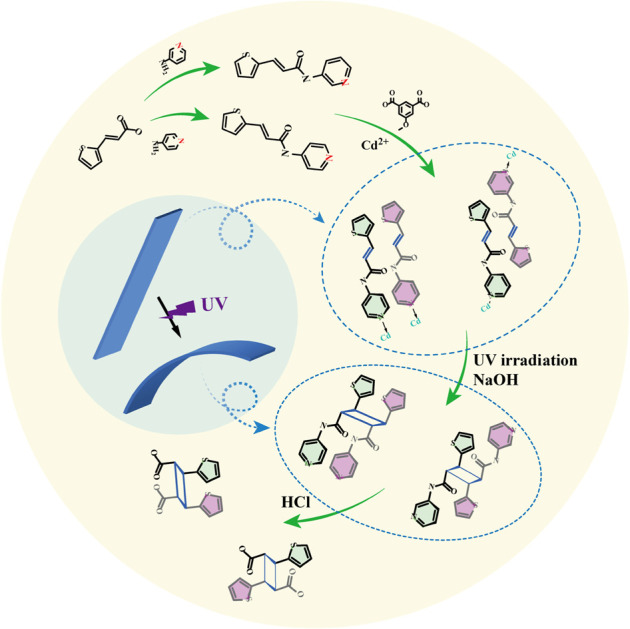

## Introduction

Stimulus-responsive materials have attracted significant attention because they can change their own molecular structure or state after receiving external stimuli such as light,^1^ heat,^2^ electricity,^3,4^ magnetism,^5^ and humidity,^6^ leading to significant changes in material properties. Among these, photoelectric materials can convert light energy into mechanical energy, realizing accurate remote controllable braking in a non-contact and non-invasive form,^7–9^ and offering great application prospects in biomedicine, soft robots, flexible electronic devices, and bionic systems.^7,10–13^ In recent years, solid-state reactions involving structural transformation have received considerable attention from researchers in the field of CPs due to their green and efficient synthesis of regionally/stereo-selectively organic molecules.^14^ The structural changes of molecular crystals, such as the isomerization of azobenzene,^7^ cyclization of diarylethenes,^15^ and [2 + 2] cycloaddition^16–19^ and [4 + 4] cycloaddition,^20,21^ may lead to driving behaviors such as jumping, splitting, bending, expansion, and contraction.^10,11,22–28^ The solid state [2 + 2] cycloaddition reactions of alkenes not only demonstrate great potential in the construction of the target cyclobutane and its derivatives,^29–33^ but photosalient (PS) phenomena may occur due to internal stress release accumulated during structural transformation.^34–36^ However, the solid-state [2 + 2] cycloaddition reaction will be constrained by several topological chemistry principles, which dictate that the distance between C=C double bonds in the reaction cannot exceed 4.2 Å and they must be arranged parallel to each other.^37^ In addition, the anisotropy of macroscopic mechanical motion was found to be closely related to the crystallinity, free volume, and molecular orientation of the photo-responsive molecules.^38,39^ Therefore, controlling the stacking of light-responsive olefins to satisfy these topological chemical requirements, as well as designing and fabricating photo-actuated systems, remains a challenge.

As metal–organic templates, metal–organic frameworks (MOFs) are constructed by metal ions or metal clusters through organic ligand coordination. In the self-assembly process, combining the rigidity of the metal and flexibility of the organic ligand, the molecular structure can be well determined and fine-tuned through design, endowing MOFs with an advantage in controlling the solid-state [2 + 2] cycloaddition reaction.^40,41^ Recently, the rapid development of organic synthesis has been achieved by using non-covalent and reversible covalent bonds.^42–45^ By combining reversible amide bonds with metal ion coordination, the functionalization and pre-assembly of olefin ligands can be achieved for intermolecular [2 + 2] photocycloaddition.^46,47^ The formation of amide bonds can reversibly functionalize an olefin ligand with a pyridine group. The pyridine nitrogen atom has a strong coordination ability and can easily coordinate with metal ions. In the presence of auxiliary carboxylic acid ligands, these can be assembled into CPs with different structures, obtaining cyclobutane derivatives with different configurations through solid [2 + 2] cycloaddition.

**Scheme 1:**
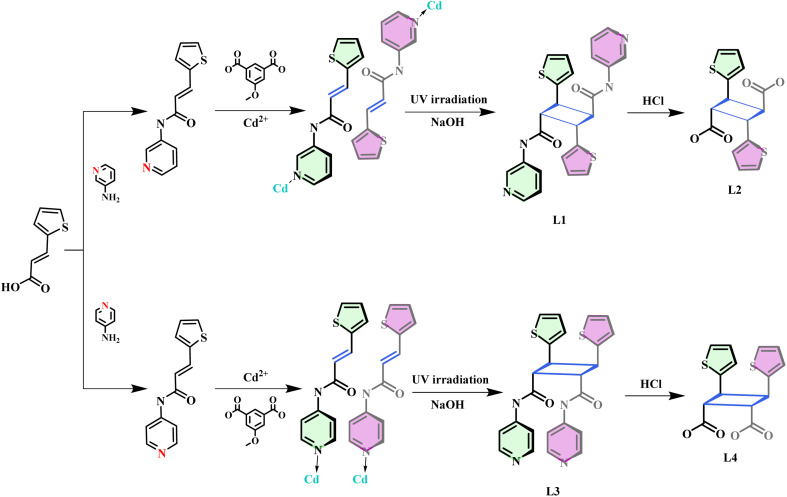
Carboxy-cyclobutane isomers were synthesized stereoselectively by a method that combined a reversible amide bond and the self-assembly of CPs by solid-state [2 + 2] cycloaddition reactions.

Because the rigid skeletons of 2D and 3D CPs exert significant limitations on the amplification of microscopic motion to crystal macroscopic motion, most photoreactive CPs reported in the literature are 1D.^19,48–50^ In this work, two pyridinamide olefin ligands, namely. (2*E*)-*N*-3-Pyridinyl-3-(2-thienyl)-2-propenamide (3-ptpa) and (2*E*)-*N*-4-Pyridinyl-3-(2-thienyl)-2-propenamide (4-ptpa), were designed by combining reversible amide bonds with pyridine N coordination site regulation. Two 1D CPs {[Cd(3-ptpa) (MeOip)·(H_2_O)_2_]·H_2_O}_*n*_ (1, MeOip = 5-methoxyisophthalic acid) and {[Cd_2_(4-ptpa)_4_(MeOip)_2_]·0.3H_2_O·0.8ACN}_*n*_ (2) were synthesized through a solvothermal reaction under the same conditions. CP1/CP2 underwent a [2 + 2] cycloaddition reaction under UV light and demonstrated photomechanical effects. In addition, through coordination bond cracking and amide bond hydrolysis, we isolated two pyridinamide cyclobutane derivatives *N*1,*N*3-di(pyridin-3-yl)-2,4-di(thiophen-2-yl)cyclobutane-1,3-dicarboxamide (L_1_) and *N*1,*N*2-di(pyridin-4-yl)-3,4-di(thiophen-2-yl)cyclobutane-1,2-dicarboxamide (L_3_), as well as two isomeric cyclobutane carboxyl derivatives 2,4-di(thiophen-2yl)cyclobutane-1,3-dicarboxylic acid (L_2_) and 3,4-di(thiophen-2-yl)cyclobutane-1,2-dicarboxylic acid (L_4_) (Scheme 1). After studying the photomechanical behavior of CP1/CP2, we fabricated an optical driving system, which combined CP1/CP2 with PVA through hydrogen bonding. This could amplify the mechanical stress and achieve the display of macroscopic kinetic behavior. This was the first example of constructing an optical driving material by self-assembly using olefin amide ligands, providing valuable clues for the preparation of CP-based optical driving materials.

## Results and discussion

### Structure description and [2 + 2] cycloaddition

**Fig. 1:**
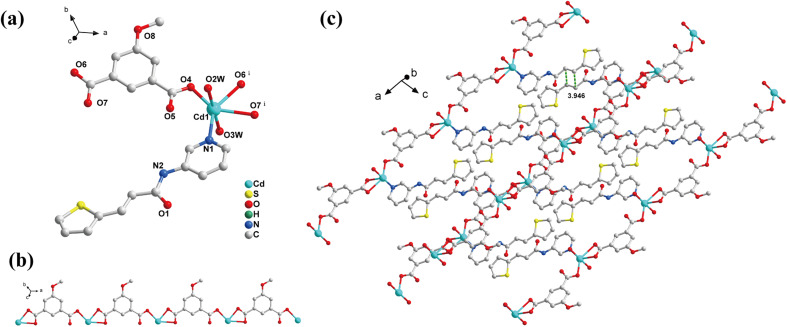
Crystal structure of 1: (a) coordination environment of the Cd^2+^ ion. Symmetry operations: (i): 1 + *x*, *y*, *z*. (b) 1D [Cd(MeOip)]_*n*_ chain. (c) 2D supramolecular structure. The hydrogen atoms and non-coordinated solvent molecules are omitted.

SCXRD analysis showed that 1 was crystallized in the triclinic system *P*ī space group with a 1D chain structure, and the asymmetric unit had a {[Cd(3-ptpa) (MeOip)·(H_2_O)_2_]·H_2_O} unit. The Cd^2+^ ion consisted of a [CdNO_5_] coordination configuration of a hexagonal twisted octahedron. The coordination atoms consisted of one N proton on 3-ptpa, five O atoms from two MeOip^2−^ ligands, and two water molecules (Fig. 1a). The MeOip^2−^ ligand adopted the μ_2-_η^1^:η^1^,η^1^ coordination mode (Scheme S3 III, Fig. S2a†), where the adjacent Cd^2+^ ions were joined by the MeOip^2−^ ions to form an infinite diffusion 1D chain extending along the *a*-axis (Fig. 1b), where the distance of the Cd^2+^ ions between the adjacent chains was 5.951 Å (Fig. S1a†).

The 3-ptpa ligands on the 1D chain between the adjacent layers were arranged head-to-tail, where the distance between the thiophene vinylolefin bond centers was 3.946 Å (Fig. 1c). This distance and orientation of the olefin pairs met the Schmidt topochemical criteria for the [2 + 2] cycloaddition reactions under UV light. In addition, the 1D CP was predicted to undergo photodimerization of the 3-ptpa ligand and transform into a 1D double-stranded stepped polymer under UV light.

The photoreactivity of 1 was studied using a custom 400 W high pressure Hg lamp photoreactor (*λ*_max_ = 365 nm), and the reaction process was detected by ^1^H-NMR spectroscopy. The ^1^H-NMR spectra showed that the cyclobutane protons gradually appeared at 4.24 and 4.51 ppm, and the olefin signal gradually disappeared from the olefin signals at 6.58 and 8.61 ppm, respectively. Using the pyridine proton labeled as (*h*′) as a reference, after 15 h of UV irradiation, the conversion rate reached 89% (Fig. S3b†).

**Fig. 2:**
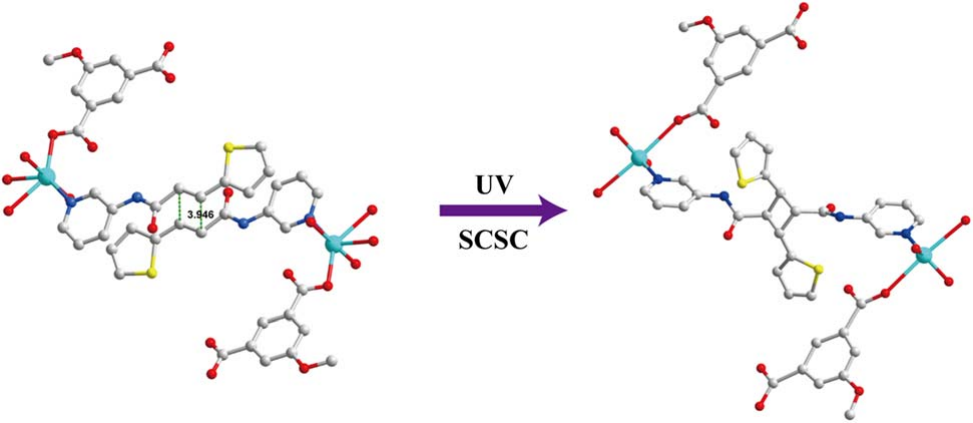
The single crystal structure of photoreactive and photoactive 1D CP1 and dimerized 1D CP1a shows the structural transformation.

A single crystal of 1 was irradiated by UV light and converted into [Cd_2_(L_1_) (MeOip)_2_(H_2_O)_4_]_*n*_ (1a) in a single-crystal to single-crystal manner (Fig. 2). Due to the weak diffraction of the 1a crystal for SCXRD analysis, the structure determination was performed by quantum mechanics calculation. This showed that 1a was in the triclinic *P*ī space group, with one [Cd_2_(L_1_) (MeOip)_2_(H_2_O)_4_] in the asymmetric unit. The central atom of 1a, namely the Cd^2+^ ion, was pentacentate, which formed a twisted triangular bipyramidal [CdNO_4_] coordination configuration with one N atom (L_1_ molecule) and four O atoms (two MeOip^2−^ and two water molecules). The acquisition of 1a confirmed the head-to-tail photodimerization of the ligand.

**Fig. 3:**
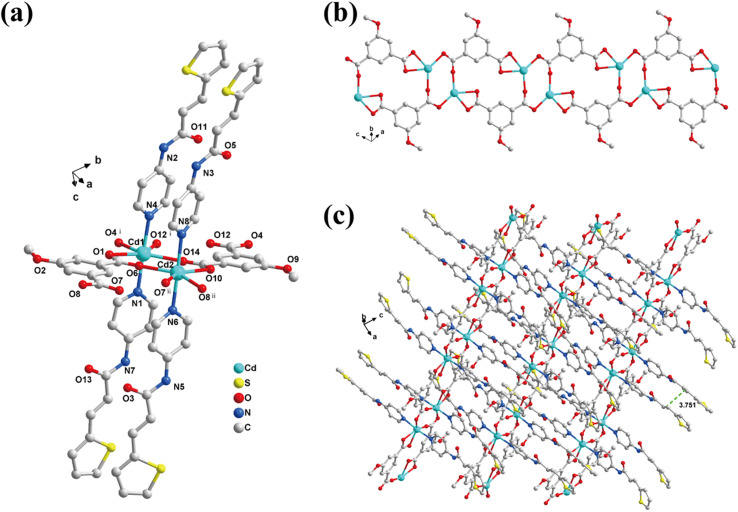
Crystal structure of 2: (a) coordination environment of the Cd^2+^ ion. Symmetry operations: (i): 1 − *x*, 1 − *y*, −*z*, (ii): −*x*, 1 − *y*, 1 − *z*. (b) 1D [Cd_2_(MeOip)_4_]_*n*_ chain. (c) 2D supramolecular structure. The hydrogen atoms and non-coordinated solvent molecules are omitted.

According to the literature, the coordination mode of the CPs changes due to the different locations of the coordination site.^51,52^ As shown in Scheme S3,† H_2_MeOip exhibited multiple coordination modes and deprotonation types.^53^ After studying the structure of 1, we aimed to control the position of pyridine N in the ligand 3-ptpa, reduce steric hindrance, and change the coordination mode between H_2_MeOip and the Cd^2+^ ions. This allowed for the bridging of the Cd^2+^ ions on the adjacent chains, shortening the distance between two Cd^2+^ ions and resulting in a structure with the olefinic ligands arranged in-phase. Therefore, we successfully synthesized a less hindered ligand, 4-ptpa, by replacing 3-aminopyridine with 4-aminopyridine. Under identical experimental conditions, we obtained another yellow block-shaped crystal {[Cd_2_(4-ptpa)_4_(MeOip)_2_]·0.3H_2_O·0.8ACN}_*n*_ (2). SCXRD analysis showed that 2 crystallized in the triclinic system *P*ī space group with a 1D chain structure. Its asymmetric unit had a {[Cd_2_(4-ptpa)_4_(MeOip)_2_]·0.3H_2_O·0.8ACN} unit, and the Cd^2+^ ion had a hexagonal twisted octahedral [CdN_2_O_4_] coordination configuration. The coordination atoms consisted of two N atoms on two 4-ptpa ligands and four O atoms on three MeOip^2−^ ligands (Fig. 3a). As expected, the MeOip ligands in 2 used the μ_3_-η^1^:η^1^,η^1^,η^1^ outer three-toothed bridged mode (Scheme S3 II, Fig. S2b†), with the non-chelating ends of the two MeOip ligands forming an anti-synchronously bridged [Cd_2_(MeOip)_4_] dimer unit that continuously diffused to form a 1D double-stranded step polymer with a distance of 3.864 Å between the adjacent Cd^2+^ ions (Fig. S1b†). In the 1D chain of 2, the adjacent 4-ptpa ligands were arranged in a head-to-head fashion, where the distance between the thiophene vinylolefin bond centers was 3.751 Å. This provided the conditions for the [2 + 2] cycloaddition reaction to occur (Fig. 3c).

The single crystal of 2 was irradiated with a 400 W high-pressure Hg (*λ*_max_ = 365 nm) lamp, and samples were obtained at regular intervals and tested by ^1^H-NMR. The ^1^H-NMR spectra showed that the cyclobutane protons gradually appeared at 4.23 and 4.50 ppm, and the olefin signal gradually disappeared from the olefin signals at 6.62 and 7.77 ppm, respectively. Using the pyridine proton labeled as (*h*′) as a reference, after 12 h of UV irradiation, the conversion rate reached 98% (Fig. S5b†).

The fitting results of C=C double bond conversion in 1/2 with UV irradiation time showed that ln(*C*/*C*_0_) had a linear relationship with irradiation time under 298 K irradiation, with *C*_0_ and *C* representing the percentage/concentration of the C=C double bond before irradiation and at any irradiation time. This demonstrated that the [2 + 2] cycloaddition reactions of 1 and 2 exhibited first-order kinetics at 298 K, with rate constants of 1.47 × 10^−1^ min^−1^ and 8.03 × 10^−2^ min^−1^, respectively (Fig. S4c and d, S5c and d†).

**Fig. 4:**
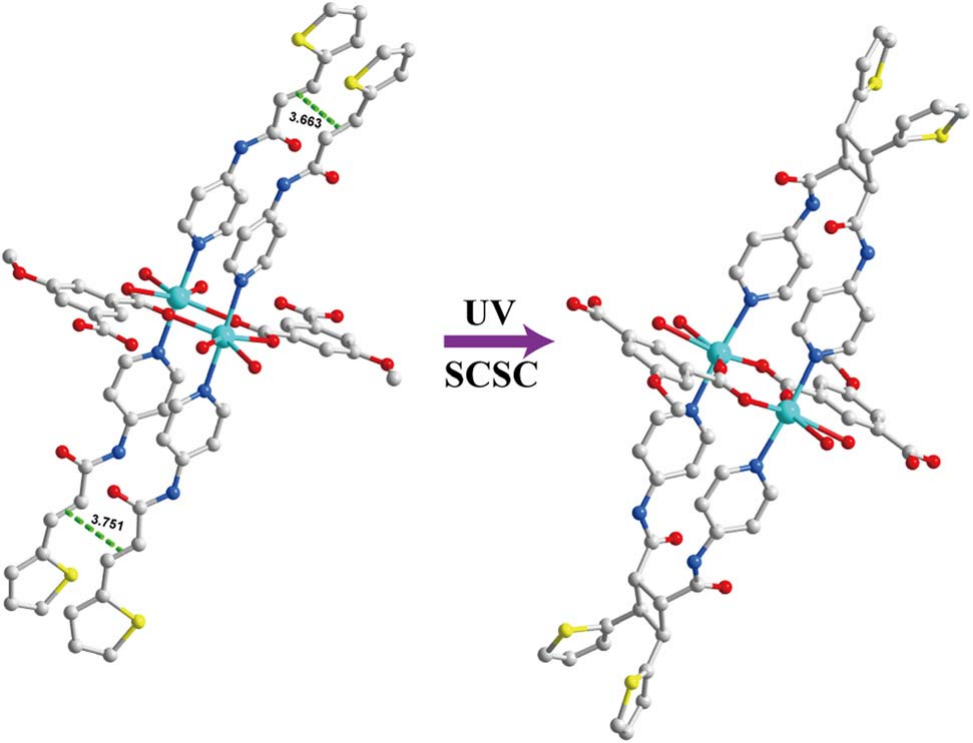
The single crystal structure of photoreactive and photoactive 1D CP2 and dimerized 1D CP2a shows the structural transformation.

The single crystal of 2 was irradiated by UV light for 12 h, and the solid-state [2 + 2] cycloaddition reaction occurred in the form of single crystal to single crystal, resulting in {[Cd(L_3_) (MeOip)]·1.25H_2_O}_*n*_ (2a) (Fig. 4). SCXRD analysis showed that 2a was also crystallized in the *P*ī space group of the triclinic system, with a {[Cd(L_3_) (MeOip)]·1.25H_2_O} unit in the asymmetric unit. The structure of 2a consisted of a one-dimensional chain structure similar to 2. The difference was that in the latter, a pair of 4-ptpa ligands was replaced by their corresponding solid-state [2 + 2] photodimerization reaction product L_3_.

### PXRD, thermal analysis and UV-VIS absorption spectrum

The resulting four crystals (1-2a) were tested by PXRD to verify their purity. By comparing the PXRD data simulated by the crystal data with the PXRD data measured by the experiment, we found that the PXRD pattern was consistent with the simulation of single crystals, indicating that the four crystals have high purity and crystallinity (Fig. S3†). TGA of the four samples was conducted from room temperature to 800 °C to examine their thermal stability. For 1 and 1a, the mass loss from the start to 158 °C and 225 °C was 10.27% and 4.26%, respectively, which was consistent with the removal of 3.5H_2_O molecules (calc., 10.53%) and 2.5H_2_O molecules (calc., 3.93%), respectively, per formula unit. 1 and 1a collapsed from 270 °C and 263 °C. respectively. For 2, the mass loss was 3.6% at 259 °C, which was consistent with the mass of one H_2_O molecule and one ACN molecule (calc., 3.7%), while for 2a, the mass loss was 2.34% at 175 °C, which was fairly consistent with the mass of one water molecule (calc., 2.34%). The dehydrated solids started to decompose at 290 °C and 223 °C respectively. These results indicated that 1-2a exhibited high thermal stability (Fig. S28†).

The solid-state optical absorbance of the ligand and synthesized 1-2a was studied using the solid-state UV-visible light absorption spectra at room temperature, indicating that 1-2a had the largest UV-vis absorption peaks at 293, 209, 291, and 210 nm (Fig. S29†). According to the solid diffuse reflection data, the Kubelka–Munk equation was used to calculate the energy band gap (*E*_g_) values of 1-2a. As shown in Fig. S30,† the *E*_g_ values of 1-2a were 3.06, 3.52, 3.10, and 3.15 eV, and the increase in *E*_g_ confirmed the conversion from 1/2 to 1a/2a.

### Separation of cyclobutane derivatives

To obtain the cyclobutane derivatives, 1a and 2a were subjected to alkaline conditions, the coordination bonds around the CP metal center were broken, and a pair of cyclobutanylamide dimer L_1_ and L_3_ molecules was separated. The amide bonds were easily reduced to carboxylic groups through a hydrolysis reaction, thus, 1a and 2a were stirred and filtered under acidic conditions, and the filtrate was condensed and returned to obtain the cyclobutanyl carboxylic dimer L_2_ and L_4_ molecules. The ^1^H-NMR, ^1^C-NMR, IR, and mass spectrometry results showed that the cyclobutane derivatives exhibited high purity (Fig. S8–S17, S23–S26†).

### Photosalient effects

**Fig. 5:**
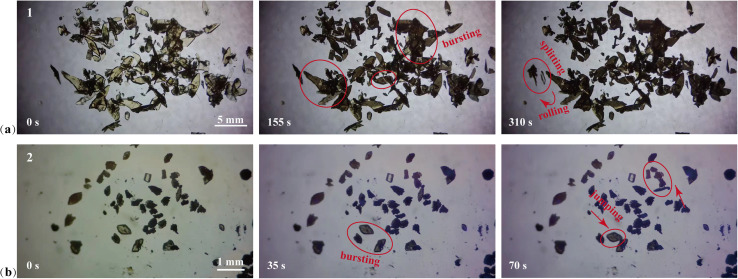
Optical images of crystals 1 (a) and 2 (b) before and after ultraviolet light irradiation.

The PS effect was observed when the single crystals of 1 and 2 were irradiated by UV light. The thicker crystals split, While the thinner crystals rolled, flipped, moved, and jumped (Fig. 5, Movies S1 and S2†). The main reason for the burst and movement of these single crystals was the stress caused by the structural transformation of the crystals in the ring addition reaction. The fragmentation of single crystal 1 occurred under ultraviolet light, and the resulting photoproduct 1a tended to form a powder, and only weak diffraction data was produced. After many attempts, the cell parameters of photoproducts 1b and 1c at 15 s and 30 s under 365 nm UV light were obtained (Fig. S33†). The transformation of 1 to 1b and 1c was mainly expansion along the *c* axis (0.41%, 1.38%), which was accompanied by expansion of the unit cell volume from 1106 Å^3^ to 1112 Å^3^ (0.45%) and 1121 Å^3^ (1.35%) (Table S6†). For single crystal 2, the unit cell volume of 2 was almost twice that of 2a due to the enhanced crystal symmetry caused by the photocyclization. The asymmetric unit in 2a could be thought of as half of the asymmetric unit in 2. The unit cell volume of 2a was accompanied by a shrinkage of about 4.32% compared to 1/2 of the unit cell volume of single crystal 2 (Table S6†). The release of stress caused by the expansion and contraction of the unit cell volume causes the fragmentation and movement of the crystal.

**Fig. 6:**
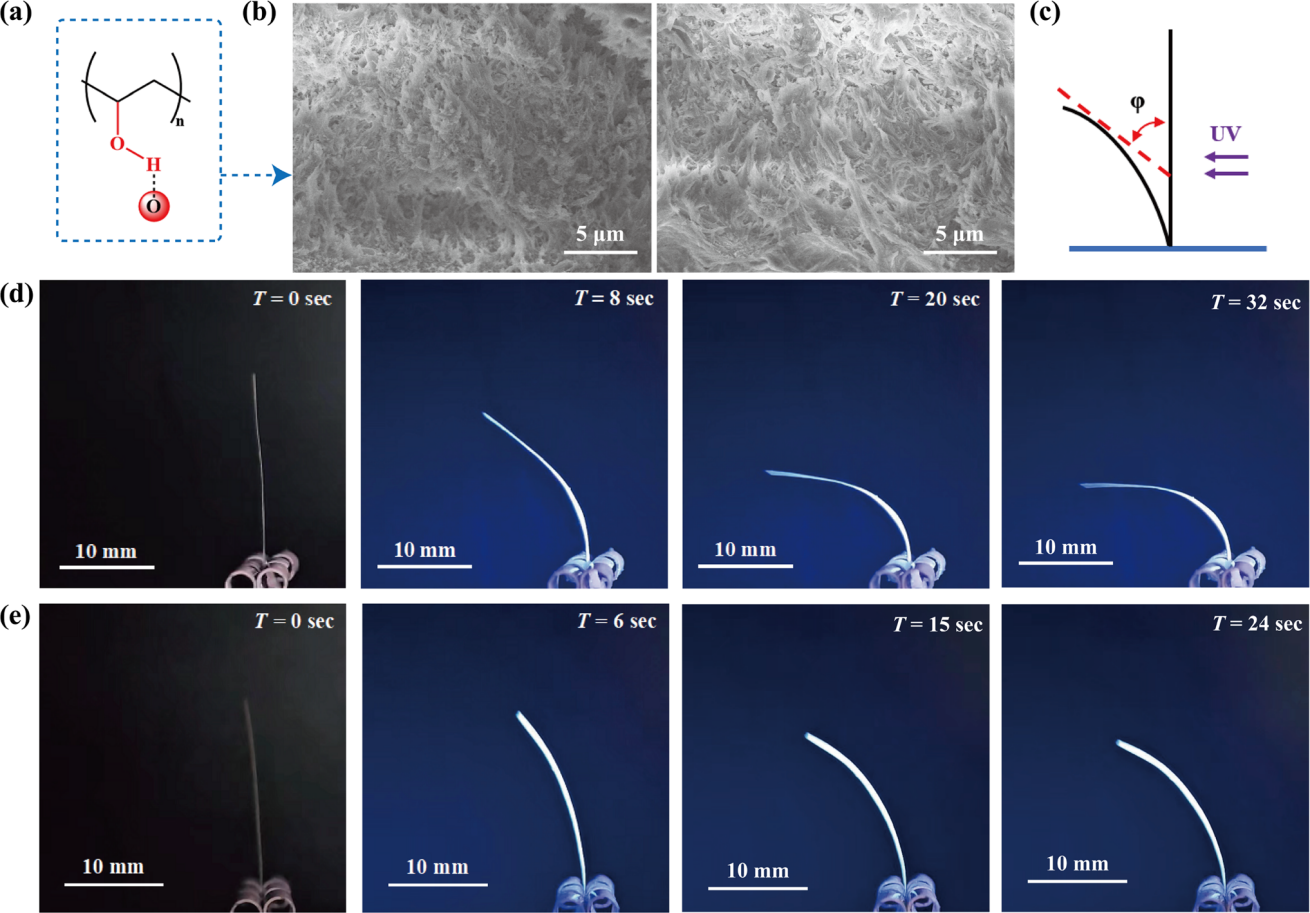
(a) Schematic diagram of hydrogen bond crosslinking networks in CPs and PVA substrates (b) scanning electron microscopy (SEM) image of the 1-PVA (left) and 2-PVA (right) membrane cross-section. (c) Indication of the bending angle *φ* in the schematic structure. Time-dependent photomechanical deformation of 1-PVA (d) and 2-PVA (e) upon exposure to 365 nm light.

In general, the inorganic and crystalline materials were brittle compared to the soft materials and were unlikely to play a useful technical role in isolation, making it still necessary to transform and amplify the microscopic changes that accompanied the photoinduced stress into macroscopic mechanical behavior in the system.^50,54^ Polyvinyl alcohol (PVA) had abundant OH groups, which could interact with the carbonyl group of the CPs by forming O–H⋯O hydrogen bonds (Fig. 6a, S27†). In this work, we employed a mixed matrix membrane strategy to combine the photoresponsive crystals of 1 and 2 with polymer PVA to enhance the photomechanical properties.^48,49,55^ The PXRD pattern of 1-PVA/2-PVA matched the combined pattern of PVA and 1/2, maintaining the crystallinity of 1/2 (Fig. S35†). The SEM images showed that the 1-PVA/2-PVA particles (after grinding) still retained the shape of the crystals (Fig. S31 and S32†). The SEM image of the surface or cross section of 1-PVA/2-PVA films and the EDS images indicated that the particles of 1 and 2 were uniformly dispersed in the PVA matrix (Fig. 6b, S31 and S32†).

To study the photomechanical properties, the composite film (0.5 × 2.0 cm) was exposed to a 365 nm UV lamp, and photomechanical deformation was recorded. As shown in Fig. 6d and S36,† when the 1-PVA film is irradiated with UV light, it rapidly backlights by ∼90° within 32 s. For 2-PVA (Fig. 6e, S37†), the backlight bending occurred under ultraviolet light, and the bending Angle was about 66° after 24 s. The photo-responsive behavior of 1-PVA/2-PVA was attributed to the photodimerization of CP1/CP2. The more pronounced bending of 1-PVA was possibly due to the fact that [2 + 2] cycloaddition of the olefin ligand in CP1 occurred between the adjacent chains, with greater energy transition than the reaction on the same chain in CP2. To further understand the kinematics and mechanism of lithographic deformation, a camera (8 K, 30 fps) was used to record a series of photomechanical deformation images. By fitting the variations of the 1-PVA/2-PVA bending angle (*φ*) with UV irradiation time (*t*), we found that the bending process of the 1-PVA/2-PVA film could be expressed by functions *φ* = 90.51(1 − exp(−*t*/7.82)) and *φ* = 67.73(1 − exp(−*t*/6.55)), respectively (Fig. S38†).^56^ For a deeper understanding of the bending deformation behaviors of membranes, atomic force microscopy (AFM) was used to probe the 1-PVA/2-PVA and 1-PVA/2-PVA surfaces irradiated by UV light. The mean square roughness of the 1-PVA surface was 182 nm, however, the mean square roughness of the 1-PVA surface after illumination was approximately 564 nm (Fig. S39†). After illumination, the mean square roughness of the 2-PVA surface changed from 151 nm before illumination to 182 nm (Fig. S40†). The roughness demonstrated by the AFM image reflected the deformation of 1/2 of the crystal, due to UV light exposure. This composite film provided an effective strategy for realizing the macroscopic amplification of micromotion, with significance in the field of mechanical microdevices and robots.

## Conclusions

In summary, we combined reversible amide bonds, pyridine N coordination site regulation, and CP self-assembly to obtain two 1D photo-responsive CPs. Photomechanical bursts, jumping and movement of the crystal were achieved through [2 + 2] cycloaddition. Two cyclobutane amide derivatives and a pair of cyclobutane carboxyl isomers were isolated through coordination bond destruction and amide bond hydrolysis. In addition, the combination of photo-responsive crystals and PVA polymers could transform photoinduced stress into controllable large-scale shape film motion, providing important clues for the design of smart material photo-actuators. Moreover, we determined that this method could also be applied to the construction of functionalized cyclobutane derivatives, and the amide bonds could also be replaced by other reversible covalent bonds, allowing us to obtain more types of cyclobutane derivatives, which is critical for organic and supramolecular synthesis.

## Data availability

All article related data can be found in ESI.†

## Author contributions

Conceptualization: Q. Zhao, L. Du, L. Wang; data curation: L. Wang, S. Qiao; formal analysis: J. Zhang, L. Wang, X. Ma, Y. Chen; writing – original draft: L. Wang, S. Qiao; writing – review & editing: Q. Zhao, L. Du, L, J. Zhang.

## Conflicts of interest

The authors declare that they have no known competing financial interests or personal relationships that could have appeared to influence the work reported in this paper.

## Supplementary Material

SC-015-D3SC06098E-s001

SC-015-D3SC06098E-s002

SC-015-D3SC06098E-s003

SC-015-D3SC06098E-s004

SC-015-D3SC06098E-s005

SC-015-D3SC06098E-s006

## References

[cit1] Deng J., Li J., Chen P., Fang X., Sun X., Jiang Y., Weng W., Wang B., Peng H. (2016). Tunable Photothermal Actuators Based on a Pre-programmed Aligned Nanostructure. J. Am. Chem. Soc..

[cit2] Phillip W. A. (2016). Thermal-energy conversion: under pressure. Nat. Energy.

[cit3] Lu C., Yang Y., Wang J., Fu R., Zhao X., Zhao L., Ming Y., Hu Y., Lin H., Tao X., Li Y., Chen W. (2018). High-performance graphdiyne-based electrochemical actuators. Nat. Commun..

[cit4] Acerce M., Akdogan E. K., Chhowalla M. (2017). Metallic molybdenum disulfide nanosheet-based electrochemical actuators. Nature.

[cit5] Erb R. M., Martin J. J., Soheilian R., Pan C., Barber J. R. (2016). Actuating Soft Matter with Magnetic Torque. Adv. Funct. Mater..

[cit6] Hu Y., Li Z., Lan T., Chen W. (2016). Photoactuators for Direct Optical-to-Mechanical Energy Conversion: From Nanocomponent Assembly to Macroscopic Deformation. Adv. Mater..

[cit7] Commins P., Karothu D. P., Naumov P. (2019). Is a Bent Crystal Still a Single Crystal?. Angew Chem. Int. Ed. Engl..

[cit8] Chen M., Yao B., Kappl M., Liu S., Yuan J., Berger R., Zhang F., Butt H. J., Liu Y., Wu S. (2019). Entangled Azobenzene-Containing Polymers with Photoinduced Reversible Solid-to-Liquid Transitions for Healable and Reprocessable Photoactuators. Adv. Funct. Mater..

[cit9] Han D. D., Zhang Y. L., Ma J. N., Liu Y. Q., Han B., Sun H. B. (2016). Light-Mediated Manufacture and Manipulation of Actuators. Adv. Mater..

[cit10] Naumov P., Chizhik S., Panda M. K., Nath N. K., Boldyreva E. (2015). Mechanically Responsive Molecular Crystals. Chem. Rev..

[cit11] Ahmed E., Karothu D. P., Naumov P. (2018). Crystal Adaptronics: Mechanically Reconfigurable Elastic and Superelastic Molecular Crystals. Angew Chem. Int. Ed. Engl..

[cit12] Ko H., Javey A. (2017). Smart Actuators and Adhesives for Reconfigurable Matter. Acc. Chem. Res..

[cit13] Ikejiri S., Takashima Y., Osaki M., Yamaguchi H., Harada A. (2018). Solvent-Free Photoresponsive Artificial Muscles Rapidly Driven by Molecular Machines. J. Am. Chem. Soc..

[cit14] Yang S. Y., Deng X. L., Jin R. F., Naumov P., Panda M. K., Huang R. B., Zheng L. S., Teo B. K. (2014). Crystallographic snapshots of the interplay between reactive guest and host molecules in a porous coordination polymer: stereochemical coupling and feedback mechanism of three photoactive centers triggered by UV-induced isomerization, dimerization, and polymerization reactions. J. Am. Chem. Soc..

[cit15] Kitagawa D., Kawasaki K., Tanaka R., Kobatake S. (2017). Mechanical Behavior of Molecular Crystals Induced by Combination of Photochromic Reaction and Reversible Single-Crystal-to-Single-Crystal Phase Transition. Chem. Mater..

[cit16] Wang M. F., Mi Y., Hu F. L., Niu Z., Yin X. H., Huang Q., Wang H. F., Lang J. P. (2020). Coordination-Driven Stereospecific Control Strategy for Pure Cycloisomers in Solid-State Diene Photocycloaddition. J. Am. Chem. Soc..

[cit17] Gong W.-J., Yang Z.-Y., Hong Y.-X., Liu D., Niu Z., Braunstein P., Lang J.-P. (2022). Tetraolefin stereospecific photodimerization and photopolymerization in coordination polymers. Sci. China: Chem..

[cit18] Wang M. F., Mi Y., Hu F. L., Hirao H., Niu Z., Braunstein P., Lang J. P. (2022). Controllable multiple-step configuration transformations in a thermal/photoinduced reaction. Nat. Commun..

[cit19] Yang Z. Y., Sang X., Liu D., Li Q. Y., Lang F., Abrahams B. F., Hou H., Braunstein P., Lang J. P. (2023). Photopolymerization-Driven Macroscopic Mechanical Motions of a Composite Film Containing a Vinyl Coordination Polymer. Angew Chem. Int. Ed. Engl..

[cit20] Nishiuchi T., Kisaka K., Kubo T. (2021). Synthesis of Anthracene-Based Cyclic pi-Clusters and Elucidation of their Properties Originating from Congested Aromatic Planes. Angew Chem. Int. Ed. Engl..

[cit21] Chen K., Wang J., Feng Y., Liu H., Zhang X., Hao Y., Wang T., Huang X., Hao H. (2021). Multiple stimuli-responsive flexible crystal with 2D elastic bending, plastic twisting and photoinduced bending capabilities. J. Mater. Chem. C.

[cit22] Hayashi S., Koizumi T. (2016). Elastic Organic Crystals of a Fluorescent pi-Conjugated Molecule. Angew Chem. Int. Ed. Engl..

[cit23] Gupta P., Karothu D. P., Ahmed E., Naumov P., Nath N. K. (2018). Thermally Twistable, Photobendable, Elastically Deformable, and Self-Healable Soft Crystals. Angew Chem. Int. Ed. Engl..

[cit24] Saha S., Desiraju G. R. (2017). Crystal Engineering of Hand-Twisted Helical Crystals. J. Am. Chem. Soc..

[cit25] Kitagawa D., Tsujioka H., Tong F., Dong X., Bardeen C. J., Kobatake S. (2018). Control of Photomechanical Crystal Twisting by Illumination Direction. J. Am. Chem. Soc..

[cit26] Seki T., Mashimo T., Ito H. (2019). Anisotropic strain release in a thermosalient crystal: correlation between the microscopic orientation of molecular rearrangements and the macroscopic mechanical motion. Chem. Sci..

[cit27] Omoto K., Nakae T., Nishio M., Yamanoi Y., Kasai H., Nishibori E., Mashimo T., Seki T., Ito H., Nakamura K., Kobayashi N., Nakayama N., Goto H., Nishihara H. (2020). Thermosalience in Macrocycle-Based Soft Crystals via Anisotropic Deformation of Disilanyl Architecture. J. Am. Chem. Soc..

[cit28] Jin M., Yamamoto S., Seki T., Ito H., Garcia-Garibay M. A. (2019). Anisotropic Thermal Expansion as the Source of Macroscopic and Molecular Scale Motion in Phosphorescent Amphidynamic Crystals. Angew Chem. Int. Ed. Engl..

[cit29] Chu Q., Duncan A. J. E., Papaefstathiou G. S., Hamilton T. D., Atkinson M. B. J., Mariappan S. V. S., MacGillivray L. R. (2018). Putting Cocrystal Stoichiometry to Work: A Reactive Hydrogen-Bonded "Superassembly" Enables Nanoscale Enlargement of a Metal-Organic Rhomboid via a Solid-State Photocycloaddition. J. Am. Chem. Soc..

[cit30] Hu F. L., Mi Y., Zhu C., Abrahams B. F., Braunstein P., Lang J. P. (2018). Stereoselective Solid-State Synthesis of Substituted Cyclobutanes Assisted by Pseudorotaxane-like MOFs. Angew Chem. Int. Ed. Engl..

[cit31] Hu F., Hao W., Mucke D., Pan Q., Li Z., Qi H., Zhao Y. (2021). Highly Efficient Preparation of Single-Layer Two-Dimensional Polymer Obtained from Single-Crystal to Single-Crystal Synthesis. J. Am. Chem. Soc..

[cit32] Claassens I. E., Barbour L. J., Haynes D. A. (2019). A Multistimulus Responsive Porous Coordination Polymer: Temperature-Mediated Control of Solid-State [2+2] Cycloaddition. J. Am. Chem. Soc..

[cit33] Zhu R.-R., Wang T., Zhao L., He L., Gao F., Du L., Zhao Q.-H. (2021). Synthesis of carboxy-cyclobutane isomers combining an amide bond and self-assembly of coordination polymers in the solid state: controlling the reaction site of [2+2] cycloaddition by introducing a substituent group. Inorg. Chem. Front..

[cit34] Medishetty R., Sahoo S. C., Mulijanto C. E., Naumov P., Vittal J. J. (2015). Photosalient Behavior of Photoreactive Crystals. Chem. Mater..

[cit35] Hatano E., Morimoto M., Imai T., Hyodo K., Fujimoto A., Nishimura R., Sekine A., Yasuda N., Yokojima S., Nakamura S., Uchida K. (2017). Photosalient Phenomena that Mimic Impatiens Are Observed in Hollow Crystals of Diarylethene with a Perfluorocyclohexene Ring. Angew Chem. Int. Ed. Engl..

[cit36] Takeda T., Ozawa M., Akutagawa T. (2019). Jumping Crystal of a Hydrogen-Bonded Organic Framework Induced by the Collective Molecular Motion of a Twisted pi System. Angew Chem. Int. Ed. Engl..

[cit37] Sassi R., Bond R. R., Cairns A., Finlay D. D., Guldenring D., Libretti G., Isola L., Vaglio M., Poeta R., Campana M., Cuccia C., Badilini F. (2017). PDF-ECG in clinical practice: a model for long-term preservation of digital 12-lead ECG data. J. Electrocardiol..

[cit38] Jiang W., Niu D., Liu H., Wang C., Zhao T., Yin L., Shi Y., Chen B., Ding Y., Lu B. (2014). Photoresponsive Soft-Robotic Platform: Biomimetic Fabrication and Remote Actuation. Adv. Funct. Mater..

[cit39] Shi Y. X., Chen H. H., Zhang W. H., Day G. S., Lang J. P., Zhou H. C. (2019). Photoinduced Nonlinear Contraction Behavior in Metal-Organic Frameworks. Chemistry.

[cit40] Chen S.-L., Mi Y., Hu F.-L., Young D. J., Lang J. (2023). Pore-Directed Solid-State Selective Photoinduced E–Z Isomerization and Dimerization within Metal–Organic Frameworks. CCS Chem..

[cit41] Wang M. F., Deng Y. H., Hong Y. X., Gu J. H., Cao Y. Y., Liu Q., Braunstein P., Lang J. P. (2023). In situ observation of a stepwise [2+2] photocycloaddition process using fluorescence spectroscopy. Nat. Commun..

[cit42] Ramamurthy V., Jockusch S., Porel M. (2015). Supramolecular Photochemistry in Solution and on Surfaces: Encapsulation and Dynamics of Guest Molecules and Communication between Encapsulated and Free Molecules. Langmuir.

[cit43] Danjou P. E., De Leener G., Cornut D., Moerkerke S., Mameri S., Lascaux A., Wouters J., Brugnara A., Colasson B., Reinaud O., Jabin I. (2015). Supramolecular assistance for the selective demethylation of calixarene-based receptors. J. Org. Chem..

[cit44] Inthasot A., Dang Thy M. D., Lejeune M., Fusaro L., Reinaud O., Luhmer M., Colasson B., Jabin I. (2014). Supramolecular assistance for the selective monofunctionalization of a calix[6]arene tris-carboxylic acid-based receptor. J. Org. Chem..

[cit45] MacGillivray L., Papefstathiou G., Friščic T., Varshney D. B., Hamilton T. D. (2005). Top. Curr. Chem..

[cit46] Elacqua E., Kaushik P., Groeneman R. H., Sumrak J. C., Bucar D. K., MacGillivray L. R. (2012). A supramolecular protecting group strategy introduced to the organic solid state: enhanced reactivity through molecular pedal motion. Angew Chem. Int. Ed. Engl..

[cit47] Boz S., Stohr M., Soydaner U., Mayor M. (2009). Protecting-group-controlled surface chemistry-organization and heat-induced coupling of 4,4'-di(tert-butoxycarbonylamino)biphenyl on metal surfaces. Angew Chem. Int. Ed. Engl..

[cit48] Chen Y., Yu C., Zhu X., Yu Q. (2023). Photomechanical effects based on a one-dimensional Zn coordination polymer crystal driven by [4 + 4] cycloaddition. Dalton Trans..

[cit49] Chen Y. R., Jia M. Z., Pan J. Q., Tan B., Zhang J. (2022). Photomechanical behavior triggered by [2 + 2] cycloaddition and photochromism of a pyridinium-functionalized coordination complex. Dalton Trans..

[cit50] Cao C., Xue X. R., Li Q. Y., Zhang M. J., Abrahams B. F., Lang J. P. (2023). Phase Transition-Promoted Rapid Photomechanical Motions of Single Crystals of a Triene Coordination Polymer. Angew Chem. Int. Ed. Engl..

[cit51] Wang H.-L., Ai J.-F., Wang Y.-F., Yu S., Li Y.-L., Zhu Z.-H., Zou H.-H. (2022). In Situ Reaction and Coordination Site Regulation to Form Binuclear Dysprosium Complexes with Different Connections and Magnetic Properties. Cryst. Growth Des..

[cit52] Swarbrook A. M., Weekes R. J., Goodwin J. W., Hawes C. S. (2022). Ligand isomerism fine-tunes structure and stability in zinc complexes of fused pyrazolopyridines. Dalton Trans..

[cit53] Fan X. X., Wang H. Y., Zhang B., Kang X. Q., Gu J. Z., Xue J. J. (2023). Six metal-organic architectures from a 5-methoxyisophthalate linker: assembly, structural variety and catalytic features. RSC Adv..

[cit54] Yue Y., Norikane Y., Azumi R., Koyama E. (2018). Light-induced mechanical response in crosslinked liquid-crystalline polymers with photoswitchable glass transition temperatures. Nat. Commun..

[cit55] Troyano J., Carne-Sanchez A., Maspoch D. (2019). Programmable Self-Assembling 3D Architectures Generated by Patterning of Swellable MOF-Based Composite Films. Adv. Mater..

[cit56] Shi Y. X., Zhang W. H., Abrahams B. F., Braunstein P., Lang J. P. (2019). Fabrication of Photoactuators: Macroscopic Photomechanical Responses of Metal-Organic Frameworks to Irradiation by UV Light. Angew Chem. Int. Ed. Engl..

