# Supplementary material for: [2 + 2] cycloaddition and its photomechanical effects on 1D coordination polymers with reversible amide bonds and coordination site regulation
Source: Chem Sci. 2024 Feb 5;15(11):3971–9. doi: 10.1039/d3sc06098e (PMC10935725; doi:10.1039/d3sc06098e)
Supplement: SC-015-D3SC06098E-s005 [file SC-015-D3SC06098E-s005.pdf]

Electronic Supplementary Material (ESI) for Chemical Science.
This journal is © The Royal Society of Chemistry 2024

Supplementary material

[2+2] cycloaddition and its photomechanical effects on 1D coordination polymers with reversible amide bonds and coordination site regulation

Lei Wang, [a] Si-Bo Qiao, [a] Yan-Ting Chen, [a] Xun Ma, [a] Wei-Ming Wei, [a] Jun Zhang, [b] Lin Du*[a] and Qi-Hua Zhao*[a]

[a] Key Laboratory of Medicinal Chemistry for Natural Resource, Ministry of Education and Yunnan Province, Yunnan Characteristic Plant Extraction Laboratory, School of Chemical Science and Technology, School of Pharmacy, Yunnan University, 650500, People's Republic of China.

[b] New Energy Photovoltaic Industry Research Center, Qinghai University, Xining 810016, People's Republic of China.

* Corresponding author.

E-mail: qhzhao@ynu.edu.cn; lindu@ynu.edu.cn;

Contents

Experimental 3

Materials and methods 3

Synthesis 3

X-ray crystallography 7

Simulation details 8

Results and Discussion 19

Crystal Structures 19

PXRD 20

NMR spectra 21

Mass spectra 29

IR spectra 32

Thermo gravimertic analysis (TGA) 38

Solid–state UV–visible absorbance spectra 39

Scanning electron microscope 40

Photosalient Effects 42

References 46

Experimental

Materials and methods

All reagents and solvents used in the experiments are commercially available and require no further purification. Elemental analysis (C, H, N) was performed using an Elemental Vario ELIII analyzer. The NMR spectroscopy was recorded on the Bruker ADVANCE III HD400 (400 MHz) spectrometer with TMS in DMSO-d_6 solution as the internal reference and chemical shifts reported in parts per million (ppm). The mass spectra were recorded on the Agilent LC-MSD TOF mass spectrometer. Infrared (IR) spectra were recorded using KBr particles (4000-400 cm^{-1}) on Thermo Fisher Scientific FTIR-Nicolet iS10. All PXRD analyses were performed using the Rigaku TTRIII-18KW automatic diffractometer (Cu Kα, 1.5418 Å), scanning from 3° to 55° with a scanning step size of 0.01°. Thermal stability studies were carried out in a nitrogen atmosphere at a heating rate of 10 °C min^{-1} on a Mettler-Toledo synchronous thermal analyzer. The morphology and energy dispersion spectroscopy (EDS) data of the composite films were obtained by NovanoSEM 450 field emission scanning electron microscope (SEM) at 10 kV. Uv diffuse reflectance spectroscopy measurements were recorded by Hitachi U-4100 spectrograph, containing an integrating sphere, with $BaSO_4$ plate as standard (100% reflectivity). Fluorescence measurements were made with a cold light F98 fluorescence spectrophotometer.

Synthesis

Synthesis of (2*E*)-*N*-3-Pyridinyl-3-(2-thienyl)-2-propenamide (3-ptpa). First, (*E*)-3(thiophen-2-yl)acrylic acid (10 mmol, 1.54 g), 30 mL of *N*, *N*-dimethylformamide and 3 mL of triethylamine were added to a 100 mL round-bottomed flask and stirred until clear. 2-(7-Azabenzotriazol-1-yl)-*N*,*N*,*N'*,*N'*-tetramethyluronium hexafluorophosphate (HATU 15 mmol, 5.7 g) was added to the above clarified solution and stirred for 3 h. After that, 3-aminopyridine (9 mmol, 0.85 g) was added to the above mixture and stirred for 2 days. Finally, the obtained crude product and anhydrous potassium carbonate (10 mmol, 1.38 g) were added to 300 mL of saturated salt water and stirred for about 30 min. Then, the sample was washed with water and dried in the oven (scheme S1). Yield: 1.79 g (86.2% based on 3-aminopyridine). Anal. Calc. for $C_{12}H_{10}N_2OS$: C, 62.60%; H,

4.35%; N, 12.17%. Found: C, 62.81%; H, 4.24%; N, 12.20%. ^{1}H-NMR (400 MHz, DMSO-d_6): δ 6.58(1H, d), 7.15(1H, dd), 7.37(1H, dd), 7.47(1H, m), 7.68(1H, dt), 7.78(1H, d), 8.13(1H, ddd), 8.27(1H, dd), 8.81(1H, d), 10.40(1H, s). ^{13}C-NMR (400 MHz, DMSO-d_6): δ 120.09, 123.70, 126.00, 128.52, 128.74, 131.61, 133.81, 135.92, 139.53, 140.71, 144.21. MS calcd for ($C_{12}H_{10}N_2OS+H^+$): 231.0514; found: 231.0580. IR (KBr, cm^{-1}): 3439(*w*), 3242(*w*), 3182(*w*), 3117(*w*), 2976(*m*), 2911(*m*), 1687(*s*), 1627(*s*), 1608(*m*), 1584(*s*), 1556(*s*), 1475(*m*), 1422(*s*), 1361(*m*), 1323(*s*), 1278(*m*), 1270(*m*), 1244(*w*), 1229(*m*), 1202(*m*), 1164(*m*), 1131(*w*), 1107(*w*), 1077(*w*), 1021(*w*), 987(*m*), 973(*m*), 928(*w*), 858(*m*), 834(*m*), 813(*m*), 790(*w*), 699(*s*), 629(*w*), 615(*w*), 577(*w*), 565(*w*), 485(*w*), 443(*w*).

Synthesis of (2*E*)-*N*-4-Pyridinyl-3-(2-thienyl)-2-propenamide (4-ptpa). The conditions for the synthesis of **4-ptpa** were similar to those for the synthesis of **3-ptpa** by replacing 3-aminopyridine with 4-aminopyridine (9 mmol, 0.85 g) (scheme S1). Yield: 1.58 g (75.9% based on 4-aminopyridine). Anal. Calc. for $C_{12}H_{10}N_2OS$: C, 62.60%; H, 4.35%; N, 12.17%. Found: C, 62.74%; H, 4.34%; N, 12.09%. ^{1}H-NMR (400 MHz, DMSO-d_6): δ 6.57(1H, d), 7.15(1H, dd), 7.49(1H, m), 7.64(2H, m), 7.69(1H, dt), 77.81(1H, d), 8.45(2H, m), 10.56(1H, s). ^{13}C-NMR (400 MHz, DMSO-d_6): δ 113.70, 120.29, 129.06, 129.54, 132.50, 135.06, 139.91, 146.40, 150.65, 164.76. MS calcd for ($C_{12}H_{10}N_2OS+H^+$): 231.0514; found: 231.0578. IR (KBr, cm^{-1}): 3398(*m*), 3241(*w*), 3160(*w*), 2921(*m*), 1694(*m*), 1625(*s*), 1596(*s*), 1508(*s*), 1420(*m*), 1366(*m*), 1333(*s*), 1274(*m*), 1228(*w*), 1203(*m*), 1159(*s*), 1043(*w*), 1001(*w*), 986(*w*), 965(*w*), 820(*m*), 697(*m*), 564(*m*), 534(*w*), 483(*w*), 442(*w*).

Synthesis of {[Cd(3-ptpa) (MeOip)·(H_2O)$_2$]·H_2O}$_n$ (1). The concentration of HNO_3 (1 M, 0.1 mL), $CdSO_4·4H_2O$ (0.01 mmol, 3 mg), 5-Methoxyisophthalic acid (0.01 mmol, 1.96 mg), **3-ptpa** (0.01 mmol, 2.3 mg) and 1 mL of ACN were successively added into a hard glass tube (15 cm in length and 7 mm in diameter). The sealed glass tube was reacted at 130 °C for 12 h and cooled to ambient temperature at a rate of 3 °C per 60 min to generate yellow block-shaped crystals, which were collected by filtration, washed with H_2O, and dried in air. Yield: 10.2 mg (86.7%, based on **3-ptpa**). Anal. Calc. for $C_{21}H_{22}N_2O_9SCd$: C, 42.65%; H, 3.72%; N, 4.73%. Found: C, 42.73%; H, 3.86%; N, 4.66%. ^{1}H-NMR (400 MHz, DMSO-d_6): δ 3.87(3H, s), 6.58(1H, d), 7.19(1H, dd), 7.55(1H, m), 7.65(2H, d), 7.75(1H, d), 7.88(1H, d), 7.98(1H, dd), 8.08(1H, t), 8.46(1H, ddd), 8.61(1H, dd), 9.30(1H, d), 11.05(1H, s). IR (KBr, cm^{-1}): 3298(*s*), 1677(*m*), 1613(*s*), 1540(*s*),

1485(*s*), 1449(*s*), 1378(*s*), 1323(*s*), 1250(*m*), 1199(*s*), 1163(*s*), 1127(*m*), 1047(*m*), 991(*w*), 963(*m*), 920(*w*), 855(*w*), 808(*m*), 779(*m*), 735(*m*), 703(*m*), 648(*m*), 615(*m*), 564(*m*), 484(*w*), 439(*w*).

Synthesis of $[Cd_2(L_1)$ $(MeOip)_2(H_2O)_4]_n$ (1a). Colorless crystals of **1** (0.1 g) were subjected to UV radiation using a UV high-pressure mercury lamp (broad-band) for 12 h to get colorless crystals of **1a** in quantitative yield (scheme S2). Anal. Calc. for $C_{42}H_{40}N_4O_{16}S_2Cd_2$: C, 43.99%; H, 3.49%; N, 4.89%. Found: C, 43.67%; H, 3.44%; N, 4.96% (note: the theoretical values from C element analysis have a certain deviation from the actual value due to the small amount of impurities that maybe generated over the photoreaction process). ^{1}H-NMR (400 MHz, DMSO-d_6): δ 3.87(4H, s), 4.24(2H, m), 4.51(2H, m), 6.91(4H, m), 7.32(2H, dd), 7.65(2.6H, d), 8.02(4H, m),8.08(1.3H, t), 8.66(4H, d), 11.61(2H, d). IR (KBr, cm^{-1}): 3432(*m*), 3301(*m*), 3069(*m*), 1720(*m*), 1596(*s*), 1557(*s*), 1518(*s*), 1449(*m*), 1426(*m*), 1373(*s*), 1323(*m*), 1258(*m*), 1209(*m*), 1160(*m*), 1128(*m*), 1051(*m*), 1016(*w*), 920(*m*), 827(*m*), 784(*m*), 726(*m*), 701(*m*), 533(*w*), 440(*w*).

Synthesis of $\{[Cd_2(4\text{-ptpa})_4(MeOip)_2]\cdot 0.3H_2O\cdot 0.8ACN\}_n$ (2). The conditions for synthesis of **2** were similar to those for synthesis of **1**, and **3-ptpa** was replaced by **4-ptpa** (0.01 mmol, 2.3 mg,). Yield: 27.4 mg (87.2%, based on **4-ptpa**). Anal. Calc. for $C_{67.5}H_{57.8}N_{8.8}O_{14.3}S_4Cd_2$: C, 51.51%; H, 3.68%; N, 7.83%. Found: C, 52.01%; H, 3.86%; N,7.74%. ^{1}H-NMR (400 MHz, DMSO-d_6): δ 3.86(3H, s), 6.62(2H, d), 7.19(2H, dd), 7.58(2H, dd), 7.64(2H, d), 7.77(2H, dt), 7.94(2H, d), 8.09(3H, m), 8.13(2H, d), 8.70(4H, d), 11.58(2H, s). IR (KBr, cm^{-1}): 3332(*m*), 3162(*w*), 3070(*w*), 3000(*w*), 1701(*s*), 1611(*s*), 1566(*s*), 1513(*s*), 1449(*m*), 1426(*s*), 1367(*s*), 1333(*s*), 1318(*m*), 1277(*m*), 1258(*m*), 1233(*w*), 1207(*s*), 1158(*s*), 1051(*m*), 1019(*m*), 982(*w*), 921(*w*), 893(*w*), 858(*w*), 825(*m*), 798(*w*), 784(*m*), 725(*m*), 703(*w*), 691(*w*), 618(*w*), 571(*m*), 530(*m*), 484(*w*), 448(*w*).

Synthesis of $\{[Cd_2(L_3)(MeOip)_2]\cdot H_2O\}_n$ (2a). Colorless crystals of **2** (0.1 g) were subjected to UV radiation using a UV high-pressure mercury lamp (broad-band) for 12 h to get colorless crystals of **2a** in quantitative yield (scheme S2). Anal. Calc. for $C_{35}H_{27.5}N_2O_{8.3}S_2Cd$: C, 53.53%; H, 3.50%; N, 3.57%. Found: C, 53.19%; H, 3.45%; N, 3.53% (note: the theoretical values from C element analysis have a certain deviation from the actual value due to the small amount of impurities that maybe generated over the photoreaction process). ^{1}H-NMR (400 MHz, DMSO-d_6): δ 3.86(3H, s), 4.23(2H, m), 4.50(2H, m), 6.91(4H, m), 7.31(2H, dd), 7.64(2H, d), 8.00(2H, d),8.04(3H, m), 8.65(4H, m), 11.60(2H, s). IR (KBr, cm^{-1}): 3288(*m*), 3069(*m*), 2990(*w*), 1721(*m*), 1613(*s*), 1597(*s*), 1555(*s*), 1518(*s*), 1449(*m*), 1426(*s*), 1374(*s*), 1329(*m*), 1258(*m*), 1212(*m*), 1158(*m*), 1126(*m*),

1052(*m*), 1017(*m*), 919(*w*), 826(*m*), 797(*m*), 725(*m*), 701(*w*), 620(*w*), 532(*w*), 441(*w*).

Separation of cyclobutanylamide dimer L_1. Aqueous solutions of **1a** (0.098 mmol, 112.28 mg), Ethylene Diamine Tetraacetic Acid (EDTA 2.5 mmol, 0.9 g) and NaOH (1 M, 40 mL) were added to a 25 mL round-bottomed flask and stirred for 3 days. By centrifugation and ultra-pure water washing, drying in the oven, the separated white powder was cyclobutanylamide dimer L_1. Yield: 32.9 mg (73.1%, based on **1a**). Anal. Calc. for $C_{24}H_{20}N_4O_2S_2$: C, 62.61%; H, 4.35%; N, 12.17%. Found: C, 62.81%; H, 4.05%; N, 12.54%. ^{1}H-NMR (600 MHz, DMSO-*d*$_6$): δ 4.01(2H, dd), 4.70(2H, dd), 6.98(4H, m), 7.30(4H, m), 7.78(2H, m), 8.20(2H, d), 8.52(2H, s), 10.22(2H, s). ^{13}C-NMR (600 MHz, DMSO-*d*$_6$): δ 36.64, 50.90, 124.02, 125.27, 125.92, 126.72, 127.36, 135.81, 141.43, 142.21, 144.69, 169.44. MS calcd for ($C_{24}H_{20}N_4O_2S_2$+H^+): 461.1028; found: 461.1094. IR (KBr, cm^{-1}): 3249(*w*), 3181(*w*), 3112(*w*), 3052(*w*), 2953(*w*), 1682(*s*), 1584(*m*), 1540(*m*), 1475(*w*), 1421(*s*), 1355(*w*), 1323(*w*), 1287(*m*), 1265(*m*), 1236(*w*), 1195(*w*), 1180(*w*), 1120(*w*), 1046(*w*), 1030(*m*), 908(*m*), 850(*m*), 799(*w*), 700(*s*), 623(*w*), 587(*w*), 557(*s*), 504(*w*).

Separation of cyclobutanylamide dimer L_3. The separation method of cyclobutanylamide dimer L_3 was similar to that of L_1, and **1a** was replaced by **2a** (0.098 mmol, 76.89 mg). Yield: 31.8 mg (70.5%, based on 2a). Anal. Calc. for $C_{24}H_{20}N_4O_2S_2$: C, 62.61%; H, 4.35%; N, 12.17%. Found: C, 63.07%; H, 4.66%; N, 12.03%. ^{1}H-NMR (600 MHz, DMSO-*d*$_6$): δ 4.03(2H, d), 4.50(2H, d), 6.88(4H, dt), 7.29(2H, d), 7.44(4H, m), 8.32(4H, m), 10.57(2H, m). ^{13}C-NMR (600 MHz, DMSO-*d*$_6$): δ 48.04, 109.26, 113.66, 125.37, 125.90, 127.09, 142.74, 145.97, 150.65, 170.22. MS calcd for ($C_{24}H_{20}N_4O_2S_2$+H^+): 461.1028; found: 461.1097. IR (KBr, cm^{-1}): 3555(*w*), 3287(*m*), 3160(*w*), 3071(*m*), 1686(*m*), 1592(*s*), 1531(*s*), 1416(*m*), 1384(*m*), 1330(*m*), 1294(*m*), 1212(*m*), 1041(*w*), 1000(*w*), 910(*w*), 827(*m*), 692(*m*), 535(*m*), 502(*w*), 452(*w*).

Separation of cyclobutanyl carboxylic acid dimer L_2. Cyclobutanylamide dimer L_1 (0.1 mmol, 46.0 mg) and HCl (36%,20 mL) were added to a 50 mL round-bottomed flask, condensed for reflux at 110 °C for 1 day, washed by centrifugation and ultra-pure water, and dried in the oven. The white powder separated was cyclobutanyl carboxylic acid dimer L_2. Yield: 22.0 mg (71.5%, based on L_1). Anal. Calc. for $C_{14}H_{12}O_4S_2$: C, 54.54%; H, 3.89%; N, 0.00%. Found: C, 54.98%; H, 3.51%; N, 0.00%. ^{1}H-NMR (400 MHz, DMSO-*d*$_6$): δ 4.01(4H, m), 6.98(4H, m), 7.38(2H, m), 12.43(2H, s). ^{13}C-NMR (400 MHz, DMSO-*d*$_6$): δ 37.04, 37.58, 49.72, 49.77, 50.07, 125.00, 125.21, 125.65, 125.88, 127.14, 127.49, 142.21, 142.24, 171.93, 172.49, 174.00. MS calcd for

($C_{14}H_{12}O_4S_2$+Na^+): 331.0075; found: 331.0063. IR (KBr, cm^{-1}): 2911(*w*), 1694(*s*), 1425(*w*), 1296(*w*), 1233(*m*), 1043(*w*), 964(*w*), 849(*w*), 705(*m*), 590(*w*), 556(*w*), 517(*w*).

Separation of cyclobutanyl carboxylic acid dimer L_4. A mixture of **2a** (0.1 mmol, 78.46 mg) and HCl (36%, 25 mL) were added to a 50 mL beamer, stirred at room temperature for 30 minutes, and then pumped and filtered. The filtrate was condensed and returned at 110 °C for 1 day, washed by centrifugation and ultra-pure water, and dried in the oven. The black powder separated was cyclobutanyl carboxylic acid dimer L_2. Yield: 22.9 mg (74.3%, based on **2a**). Anal. Calc. for $C_{14}H_{12}O_4S_2$: C, 54.54%; H, 3.89%; N, 0.00%. Found: C, 54.46%; H, 3.67%; N, 0.00%. ^{1}H-NMR (400 MHz, DMSO-d_6): δ 3.70(2H, m), 4.32(2H, m), 6.85(4H, m), 7.24(2H, dd), 12.56(2H, s). ^{13}C-NMR (400 MHz, DMSO-d_6): δ 41.14, 45.56, 125.32, 125.82, 127.11, 142.45, 173.53. MS calcd for ($C_{14}H_{12}O_4S_2$+Na^+): 331.0075; found: 331.0068. IR (KBr, cm^{-1}): 3192(*w*), 1744(*s*), 1696(*m*), 1618(*w*), 1413(*m*), 1285(*w*), 1245(*m*), 1196(*m*), 1177(*m*), 1137(*w*), 1043(*w*), 848(*w*), 801(*w*), 695(*m*), 598(*w*), 521(*w*), 500(*w*), 480(*w*), 459(*w*), 440(*w*).

Preparation of artificial photoactuator membrane 1-PVA. Grind **CP1** with a mortar and pestle to a uniform powder for about 30 minutes. The powder (200 mg) was dispersed in 4 mL ethanol and treated with ultrasonic bath for 5 h. Dry in a vacuum oven at 60 °C to obtain a uniform powder. The 5 g PVA solution (5 wt%) was then mixed with the **CP1** powder and stirred at 70 °C overnight to obtain a uniform solution. The composite film **1-PVA** was obtained by coating the mixture on a glass plate and drying it in an oven at 80 °C.

Preparation of artificial photoactuator membrane 2-PVA. The preparation method of the artificial photoactuator film **2-PVA** was similar to that of **1-PVA**, and **CP1** was replaced by **CP2** (200 mg).

X-ray crystallography

A selected single crystal of suitable size was mounted on a glass capillary tube for X-ray diffraction analysis. Crystallographic data were collected using graphite monochromatic Mo-Kα radiation (λ=0.71073 Å) and the ω-scanning techniques on a Bruker Smart AXS CCD diffractometer[1]. The SADABS program was used to modify the experience absorption. In each case, the structure was solved using the SHELXL software package and the SHELX-2014 program was

refined by the F^2-based full matrix least square method[2, 3]. All non-hydrogen atoms were located in a different Fourier synthesis and finally refined with anisotropic thermal parameters. Table S1 lists the crystallographic data and details of data collection and structure refinement for **1-2a**. Tables S2 and S3 list the selected key lengths and angles for **1-2a**, respectively. CCDC: **1** is 2296093, **2** is 2296091, **2a** is 2296092.

Simulation details

The data of powder x-ray diffraction were recorded on Bruker D8 advance diffractometer (40 kV, 40 mA) with step of 0.01 degree from 3° to 55° at room temperature, using graphite monochromator and Cu-Kα radiation (λ=1.5418 Å). The positions of the peaks were picked up from the diffraction pattern excluding the weak peaks agreeing with the reactant, they were further applied to index and yield the unit cell parameters by TOPAS[4, 5] program, subsequently an empty cell was constructed according to the enforced unit cell parameters, and the atoms were added to it and refined to find their ideal positions by TD-DFT method using GGA-PBE function. The final structure was checked through comparing the x-ray diffraction pattern between the experimental line and theoretical one (figure S3), their positons matched each other well and revealed the correction for the final structure.

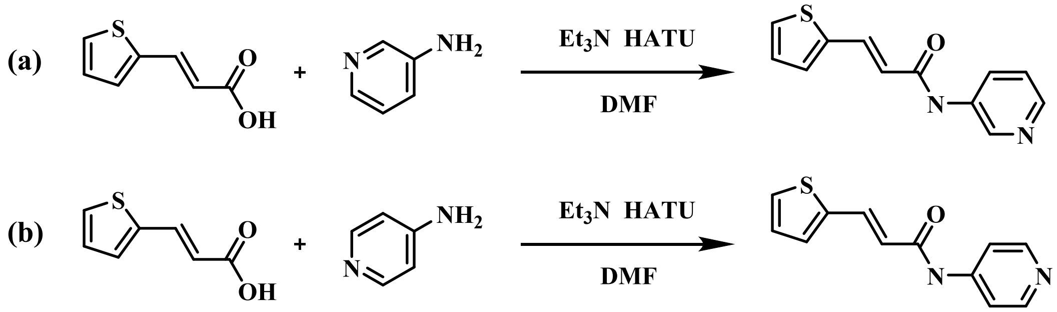

Scheme S1 Synthesis of **3-ptpa** (a) and **4-ptpa** (b).

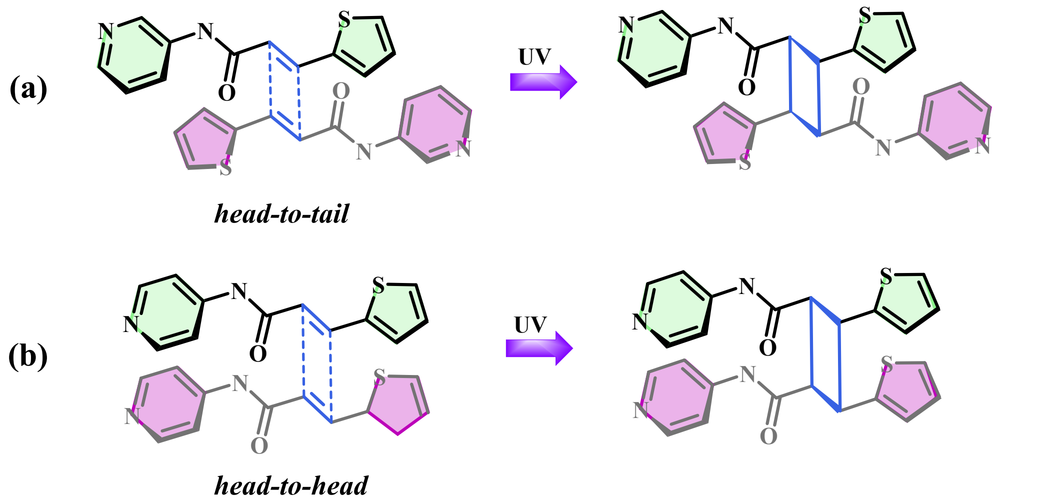

Scheme S2 The *head-to-tail* arrangement of **3-ptpa** ligands (a) and the *head-to-head* arrangement of **4-ptpa** ligands (b) result in different cycloaddition of cyclobutane derivatives under ultraviolet light.

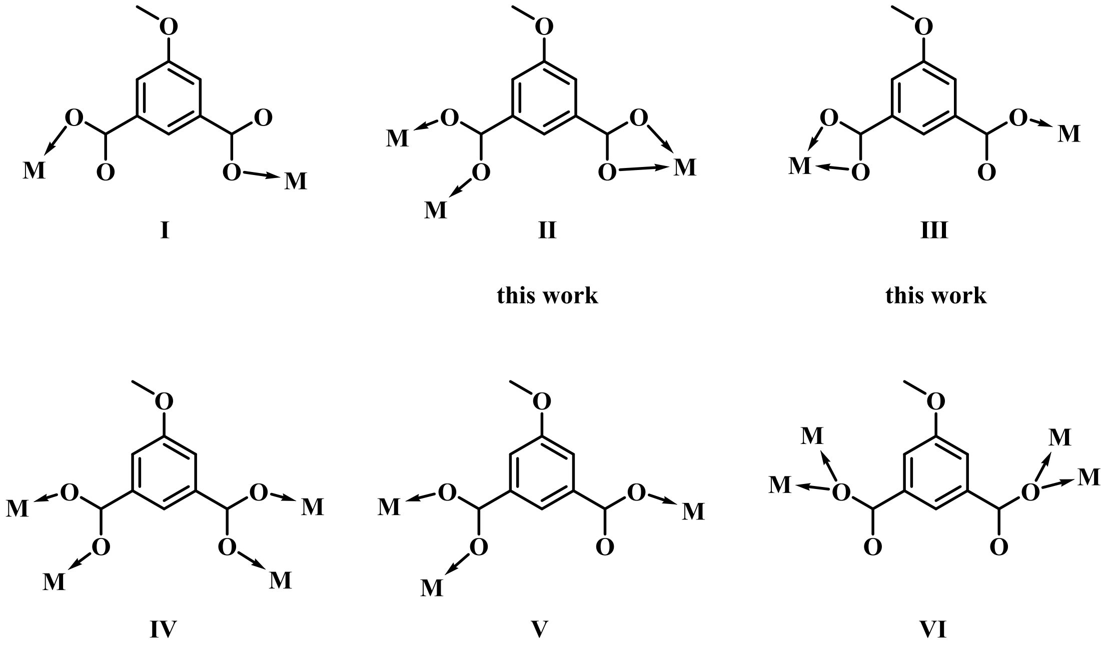

Scheme S3 Coordination pattern of ligand H_2MeOip.

Table S1 Crystallographic data and structure refinement summary for **1-2a**.

**Complex**	**1**	**2**	**2a**
chemical formula	$C_{38}H_{36}Cd_2N_8Cl_2SO_8$	$C_{66}H_{52}Cd_2N_8O_{14}S_4 \cdot 0.75(C_2H_3N) \cdot 0.25(H_2O)$	$C_{33}H_{26}CdN_4O_7S_2 \cdot 1.25(H_2O)$
molecular weight	590.86	1572.51	784.61
crystal system	Triclinic	Triclinic	Triclinic
space group	$P\bar{1}$	$P\bar{1}$	$P\bar{1}$
a (Å)	10.2530 (4)	12.5971 (5)	10.5185 (18)
b (Å)	10.3656 (5)	16.0044 (8)	10.7250 (18)
c (Å)	12.3264 (6)	16.5980 (8)	14.858 (3)
α (deg)	93	87.861 (2)	93.035 (5)
β (deg)	101	89.132 (1)	95.697 (6)
γ (deg)	118	85.016 (2)	106.480 (5)
V (Å^3)	1106.38 (9)	3331.1 (3)	1593.6 (5)
Z	2	2	2
D_{calcd} (g·cm^{-3})	1.774	1.568	1.635
μ (Mo$K\alpha$) (mm^{-1})	1.139	0.84	0.875
F (000)	596	1596	795
reflections collected/unique	48654	125248	39230
R_{int}	0.040	0.074	0.053

data/restraints/parameters	5517/0/313	16644/461/923	7941/542/526
GOF on F_2	1.054	1.041	1.102
R_1/wR_2 [$I > 2\sigma(I)$]	0.0224, 0.0566	0.0459, 0.1055	0.0476, 0.1022
R_1/wR_2 [all data]	0.0254, 0.0581	0.0712, 0.1195	0.0667, 0.1156

[a]$R_1 = \sum||F_o|-|F_c||/\sum|F_o|$, [b]$wR^2 = [\Sigma w(F_o^2 - F_c^2)^2 / \Sigma w(F_o^2)^2]^{1/2}$

Table S2 Selected Bond Distances (Å) and Angles (deg) for **1**.

**1**			
Cd1—O6^{i}	2.325 (13)	C14—C13	1.504 (2)
Cd1—O4	2.300 (13)	C3—H3	0.950
Cd1—O3	2.298 (13)	C3—C4	1.425 (2)
Cd1—O2	2.348 (14)	C3—C2	1.433 (3)
Cd1—O7^{i}	2.527 (13)	C15—H15	0.950
Cd1—N1	2.295 (15)	C15—C20	1.390 (2)
S1—C4	1.719 (19)	C18—C17	1.503 (2)
S1—C1	1.702 (2)	C8—C10	1.397 (2)
O6—C18	1.265 (2)	C8—C9	1.391 (3)
O4—C13	1.270 (2)	C19—H19	0.950
O3—H3A	0.872	C19—C17	1.393 (2)
O3—H3B	0.871	C19—C20	1.393 (2)
O8—C20	1.382 (2)	C10—H10	0.950
O8—C21	1.442 (2)	C12—H12	0.950
O5—C13	1.253 (2)	C12—C11	1.380 (3)
O9—H9A	0.870	C5—H5	0.950
O9—H9B	0.870	C5—C4	1.447 (3)
O2—H2A	0.872	C5—C6^{ii}	3.945 (3)
O2—H2B	0.872	C5—C6	1.341 (3)
O7—C18	1.264 (2)	C11—H11	0.950
O1—C7	1.223 (2)	C11—C9	1.388 (3)
N1—C10	1.334 (2)	C9—H9	0.950
N1—C12	1.344 (2)	C21—H21A	0.980
N2—H2	0.880	C21—H21B	0.980
N2—C8	1.401 (2)	C21—H21C	0.980
N2—C7	1.370 (2)	C7—C6	1.477 (3)
C16—H16	0.950	C1—H1	0.950
C16—C14	1.393 (2)	C1—C2	1.357 (3)
C16—C17	1.395 (2)	C2—H2C	0.950
C14—C15	1.398 (2)	C6—H6	0.950

Symmetry codes: (i) i: 1+*x*, *y*, *z*.

Table S3 Selected Bond Distances (Å) and Angles (deg) for **1a**.

**1a**			
Cd1—O4	2.023	C25—H26	1.082
Cd1—O8	2.017	C25—C27	1.393
Cd1—N12	2.120	C27—H28	1.079
Cd1—O90	2.571	C30—C35	1.513
Cd1—O92	2.601	C31—H32	1.077
Cd1—O3^{i}	3.143	C31—C33	1.376
S2—C29	1.720	C33—H34	1.078
S2—C31	1.702	C35—H36	1.110
O4—H5	0.990	C35—C23^{ii}	1.542
O4—H6	0.990	O90—C98	1.350
O8—H9	0.990	O91—C102	1.437
O8—H10	0.990	O91—C103	1.427
O11—C30	1.216	O92—C98	1.343
N12—C19	1.363	C93—H94	0.995
N12—C21	1.361	C93—C95	1.747
N13—H14	1.045	C93—C101	1.762
N13—C17	1.448	C95—C96	1.461
N13—C30	1.380	C95—C18	1.824
C15—H16	1.083	C96—H97	1.054
C15—C29	1.377	C96—C102	1.480
C15—C33	1.362	C98—C101	1.851
C17—C19	1.410	C99—H100	1.030
C17—C27	1.407	C99—C101	1.443
C19—H20	1.080	C99—C102	1.505
C21—H22	1.080	C103—H104	1.111
C21—C25	1.393	C103—H105	1.109
C23—H24	1.115	C103—H106	1.111
C23—C29	1.507	O3—C18	1.506
C23—C35	1.540	O3—Cd1^{iii}	3.143

C23—C35ii	1.542	O7—C18	1.269

Symmetry codes: (i) *x*-1, *y*, *z*; (ii) -*x*, -*y*+1, -*z*+1; (iii) *x*+1, *y*, *z*.

Table S4 Selected Bond Distances (Å) and Angles (deg) for **2**.

**2**			
Cd1—O1	2.341 (2)	C38—C42	1.391 (5)
Cd1—O4i	2.397 (2)	C40—C56	1.393 (5)
Cd1—O12i	2.448 (2)	C40—C58	1.389 (5)
Cd1—O14	2.335 (2)	C44—H44	0.950
Cd1—N1	2.276 (3)	C50—H50	0.950
Cd1—N4	2.279 (3)	C50—C58	1.385 (5)
Cd2—O6	2.295 (2)	C52—C11	1.472 (5)
Cd2—O8ii	2.338 (2)	C54—H54	0.950
Cd2—O10	2.276 (2)	C54—C56	1.380 (5)
Cd2—O7ii	2.460 (2)	C56—H56	0.950
Cd2—N6	2.303 (3)	C58—H58	0.950
Cd2—N8	2.300 (3)	C60—H60	0.950
Cd2—C48ii	2.752 (3)	C60—C3	1.392 (6)
S1—C19	1.737 (4)	C60—C13	1.374 (5)
S1—C61	1.721 (5)	C62—H62	0.950
S4—C43	1.700 (5)	C62—C5	1.378 (6)
S4—C2	1.712 (6)	C64—C66	1.467 (5)
S2—C13	1.751 (4)	C66—H66	0.950
S2—C63	1.717 (7)	C66—C17	1.335 (5)
S3—C53	1.724 (4)	C33—H33	0.950
S3—C65	1.716 (6)	C33—C39	1.470 (5)
O1—C18	1.253 (4)	C33—C23	1.342 (5)
O4—C20	1.268 (4)	C17—H17	0.950
O6—C18	1.261 (4)	C17—C53	1.441 (5)
O8—C48	1.276 (4)	C35—C45	1.476 (5)
O10—C14	1.259 (4)	C9—H9	0.950
O12—C20	1.248 (4)	C9—C43	1.432 (5)
O14—C14	1.256 (4)	C9—C45	1.340 (5)

O7—C48	1.250 (4)	C37—H37	0.950
O2—C46	1.372 (4)	C37—C11	1.341 (5)
O2—C25	1.418 (5)	C37—C13	1.440 (5)
O9—C42	1.375 (4)	C19—C23	1.444 (6)
O9—C57	1.433 (5)	C19—C47	1.374 (6)
N1—C51	1.307 (5)	C5—H5A	0.950
N1—C27	1.305 (5)	C41—H41	0.950
N4—C16	1.343 (4)	C41—C21	1.385 (6)
N4—C36	1.343 (4)	C21—H21	0.950
O5—C52	1.218 (4)	C43—C55	1.385 (6)
N6—C62	1.324 (5)	C11—H11	0.950
N6—C41	1.327 (5)	C45—H45	0.950
O11—C64	1.222 (4)	C23—H23	0.950
N8—C50	1.338 (5)	C47—H47	0.950
N8—C54	1.339 (5)	C47—C29	1.389 (7)
O3—C39	1.221 (4)	C3—H3A	0.950
N2—H2	0.880	C3—H3B	0.950
N2—C22	1.389 (4)	C3—C63	1.385 (8)
N2—C64	1.371 (4)	C3—C63A	1.340 (2)
C1—H1	0.950	C49—H49	0.950
C1—C6	1.400 (4)	C49—C53	1.361 (6)
C1—C12	1.391 (4)	C49—C15	1.397 (6)
C4—H4	0.950	C25—H25A	0.980
C4—C8	1.393 (5)	C25—H25B	0.980
C4—C10	1.403 (4)	C25—H25C	0.980
N5—H5	0.880	C51—H51	0.950
N5—C32	1.386 (5)	C51—C7	1.389 (6)
N5—C39	1.370 (5)	C13—S2A	1.728 (6)
N3—H3	0.880	C53—S3A	1.865 (7)
N3—C40	1.386 (5)	C27—H27	0.950
N3—C52	1.390 (5)	C27—C59	1.382 (6)
C6—C20	1.501 (4)	C55—H55	0.950

C6—C38	1.388 (5)	C55—C31	1.357 (7)
O13—C35	1.216 (4)	C7—H7A	0.950
C8—C18	1.513 (4)	C57—H57A	0.980
C8—C26	1.395 (5)	C57—H57B	0.980
N7—H7	0.880	C57—H57C	0.980
N7—C30	1.388 (4)	C29—H29	0.950
N7—C35	1.387 (4)	C29—C61	1.366 (8)
C10—C28	1.384 (5)	C59—H59	0.950
C10—C48	1.499 (4)	C15—H15	0.950
C12—C14	1.508 (4)	C15—H15A	0.950
C12—C34	1.398 (5)	C15—C65	1.346 (8)
C16—H16	0.950	C15—C65A	1.450 (2)
C16—C24	1.374 (5)	C61—H61	0.950
C22—C24	1.390 (5)	C31—H31	0.950
C22—C44	1.398 (4)	C31—C2	1.340 (9)
C24—H24	0.950	C63—H63	0.950
C26—H26	0.950	C2—H2A	0.950
C26—C46	1.391 (4)	C65—H65	0.950
C28—H28	0.950	S3A—C65A	1.790 (2)
C28—C46	1.387 (5)	C65A—H65A	0.950
C30—C7	1.360 (5)	C63A—H63A	0.950
C30—C59	1.359 (6)	C63A—S2A	1.760 (2)
C32—C5	1.381 (5)	C69—N68	1.162 (9)
C32—C21	1.375 (5)	C69—C67	1.421 (9)
C34—H34	0.950	C67—H67A	0.980
C34—C42	1.389 (4)	C67—H67B	0.980
C36—H36	0.950	C67—H67C	0.980
C36—C44	1.385 (5)	O67A—H67D	0.870
C38—H38	0.950	O67A—H67E	0.870

Symmetry codes: (i) 1-*x*, 1-*y*, -*z*; (ii) -*x*, 1-*y*, 1-*z*.

Table S5 Selected Bond Distances (Å) and Angles (deg) for **2a**.

**2a**

Cd1—O4	2.306 (3)	C13—C10	1.378 (6)
Cd1—O2^{i}	2.356 (3)	C12—H12	0.950
Cd1—O3^{i}	2.500 (3)	C12—C11	1.379 (5)
Cd1—O5^{ii}	2.321 (3)	C10—H10	0.950
Cd1—N2^{ii}	2.306 (3)	C16—C17	1.516 (5)
Cd1—N1	2.279 (3)	C33—H33	0.950
S1—C19	1.722 (8)	C33—C32	1.391 (5)
S1—C22	1.736 (6)	C27—H27	1.000
S2—C26	1.639 (6)	C27—C26	1.495 (6)
S2—C25	1.709 (13)	C27—C18	1.579 (6)
C23—H23	0.950	C27—C28	1.550 (5)
C23—C26	1.421 (17)	C32—H32	0.950
C23—C24	1.410 (2)	C11—H11	0.950
O4—C9	1.263 (4)	C26—S2A	1.681 (6)
O2—C8	1.278 (4)	C26—C23A	1.437 (19)
O1—C3	1.375 (4)	C19—C18	1.487 (6)
O1—C1	1.430 (5)	C29—C28	1.521 (5)
O3—C8	1.240 (4)	C18—H18	1.000
O5—C9	1.252 (5)	C18—H18A	1.000
N2—C34	1.350 (5)	C18—C17	1.552 (5)
N2—C33	1.343 (5)	C18—C19A	1.488 (15)
N3—H3	0.880	C17—H17	1.000
N3—C14	1.407 (5)	C17—C28	1.591 (6)
N3—C16	1.377 (5)	C28—H28	1.000
N1—C10	1.339 (5)	C22—H22	0.950
N1—C11	1.338 (5)	C22—C21	1.352 (8)
O7—C29	1.211 (5)	C1—H1A	0.980
N4—H4	0.880	C1—H1B	0.980
N4—C31	1.394 (5)	C1—H1C	0.980
N4—C29	1.373 (5)	C21—H21	0.950
O6—C16	1.219 (5)	C25—H25	0.950
C20—H20	0.950	C25—C24	1.344 (12)

C20—C19	1.321 (12)	C24—H24	0.950
C20—C21	1.396 (13)	S2A—C25A	1.686 (16)
C6—H6	0.950	C23A—H23A	0.950
C6—C5	1.395 (5)	C23A—C24A	1.530 (3)
C6—C7	1.397 (5)	C25A—H25A	0.950
C5—C4	1.405 (5)	C25A—C24A	1.344 (14)
C5—C9	1.518 (5)	C24A—H24A	0.950
C7—C2	1.389 (5)	O8—H8A	0.870
C7—C8	1.507 (5)	O8—H8B	0.870
C4—H4A	0.950	O8—O9	1.318 (14)
C4—C3	1.383 (5)	O9—H9A	0.870
C2—H2	0.950	O9—H9B	0.870
C2—C3	1.397 (5)	S1A—C19A	1.72 (2)
C31—C35	1.399 (5)	S1A—C22A	1.727 (18)
C31—C32	1.399 (5)	C20A—H20A	0.950
C35—H35	0.950	C20A—C19A	1.32 (2)
C35—C34	1.378 (5)	C20A—C21A	1.42 (2)
C14—C13	1.389 (5)	C22A—H22A	0.950
C14—C12	1.395 (5)	C22A—C21A	1.350 (18)
C34—H34	0.950	C21A—H21A	0.950
C13—H13	0.950		

Symmetry codes: (i) 1-*x*, *y*, *z*; (ii) 1-*x*, -*y*, -*z*.

Results and Discussion

Crystal Structures

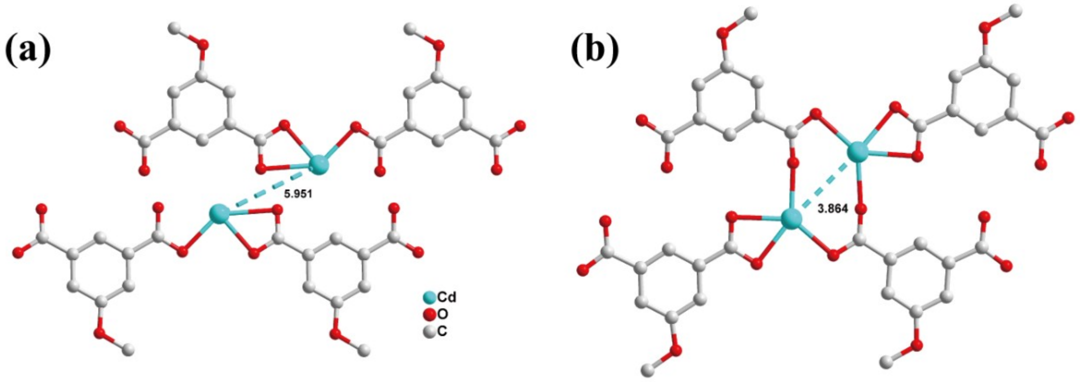

Fig. S1 Cd···Cd distance and component of the 1D carboxyl chain in **1** (a) and **2** (b).

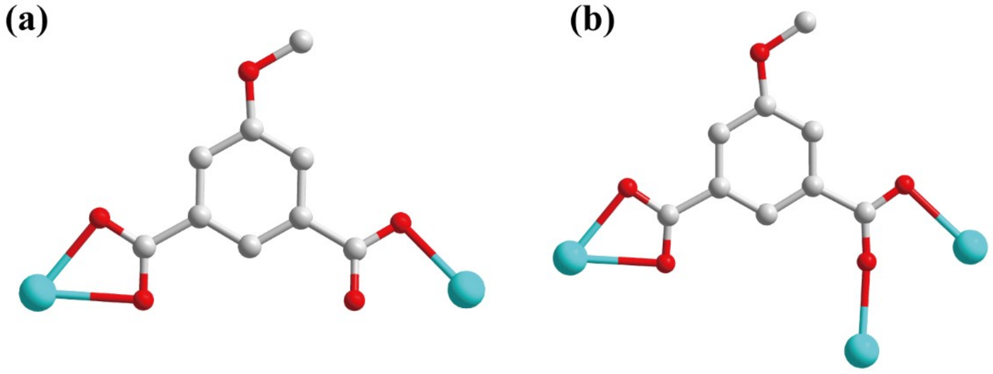

μ_2-η^1:η^1,η^1

μ_3-η^1:η^1,η^1,η^1

Fig. S2 Coordination modes of the carboxyl ligands in **1** (a) and **2** (b).

PXRD

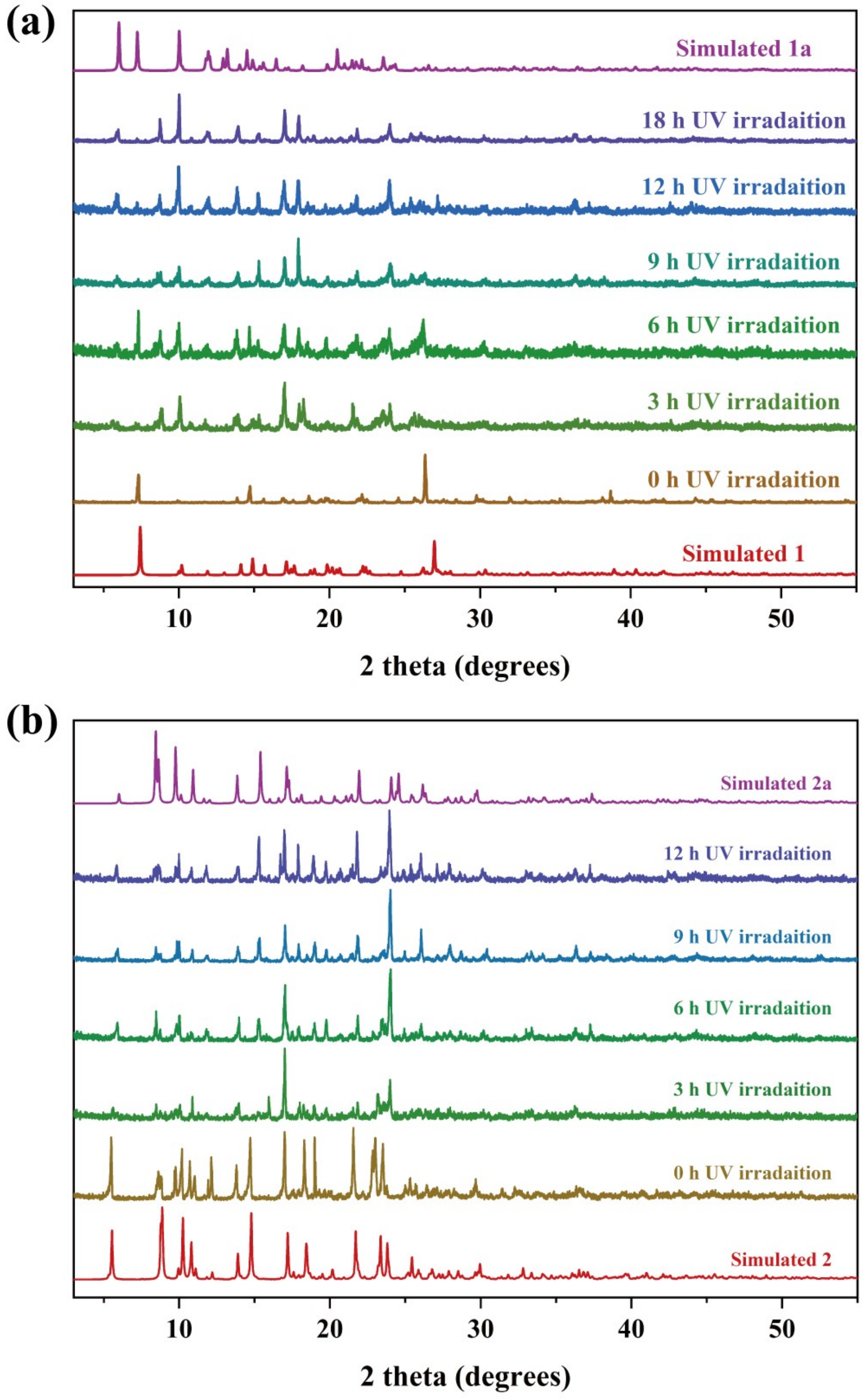

Fig. S3 PXRD patterns of **1**–**2a**.

NMR spectra

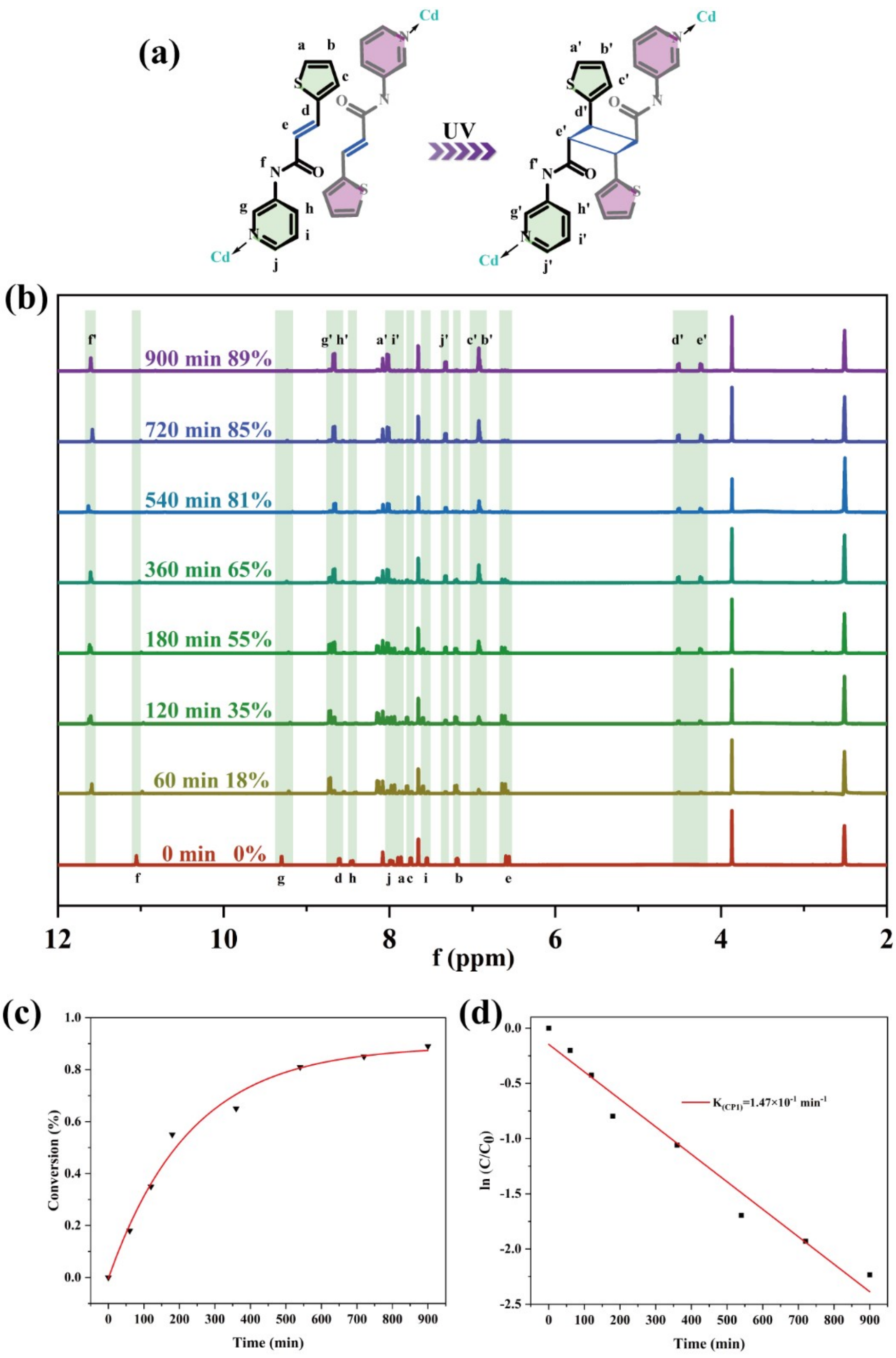

Fig. S4 The ^{1}H-NMR spectra of **1** after UV light (λ = 365 nm) irradiation at 298 K (b) for different time (400 MHz, DMSO-d_6) and the alignment of **3-ptpa** ligands before and after the photocycloaddition reaction (a). Plots of conversion of **3-ptpa** in **1** versus irradiation time based on ^{1}H-NMR result (c) and corresponding fitting of kinetic rate at 298 K of **1** (d).

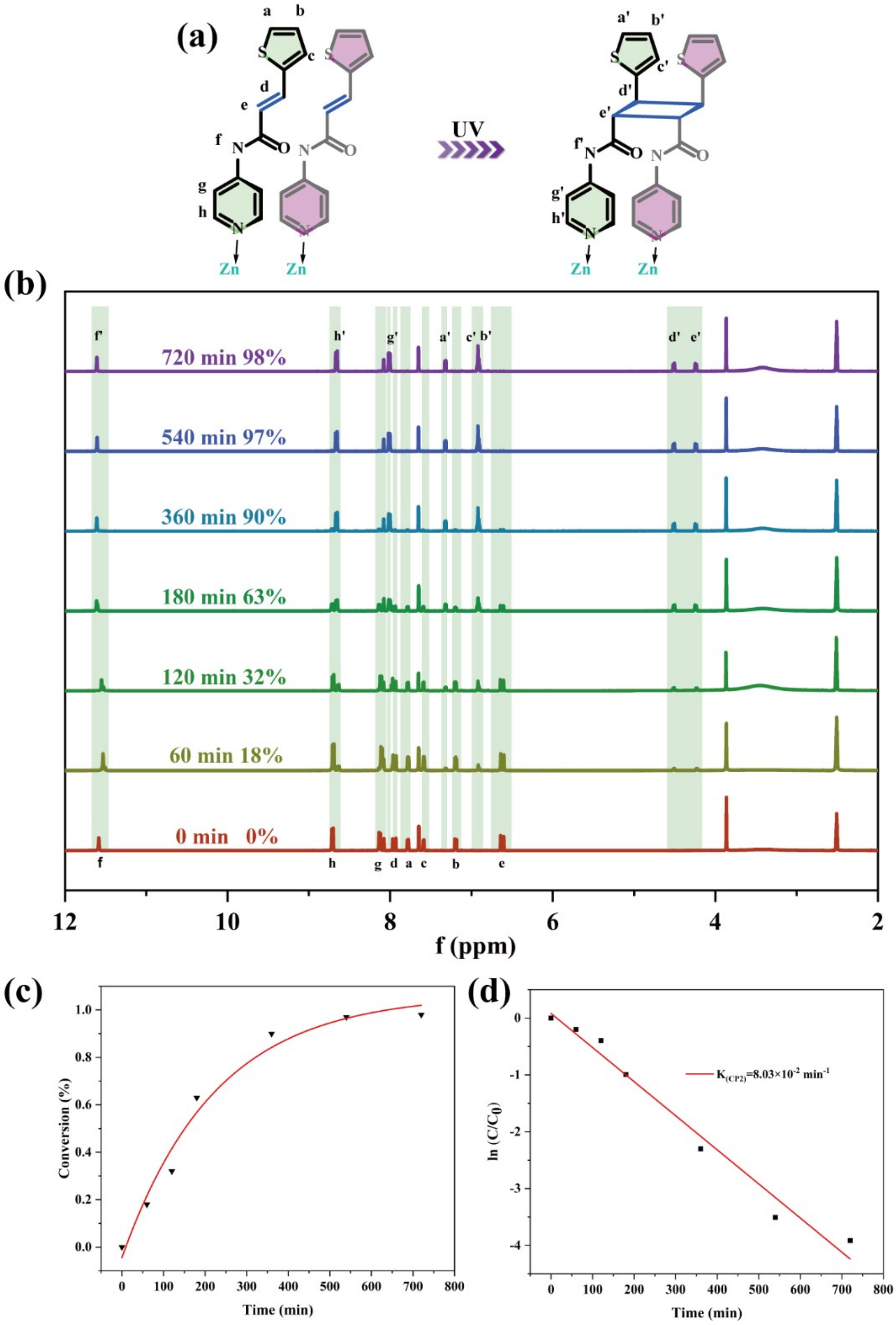

Fig. S5 The ^{1}H-NMR spectra of **2** after UV light (λ = 365 nm) irradiation at 298 K (b) for different time (400 MHz, DMSO-d_6) and the alignment of **4-ptpa** ligands before and after the photocycloaddition reaction (a). Plots of conversion of **4-ptpa** in **2** versus irradiation time based on ^{1}H-NMR result (c) and corresponding fitting of kinetic rate at 298 K of **2** (d).

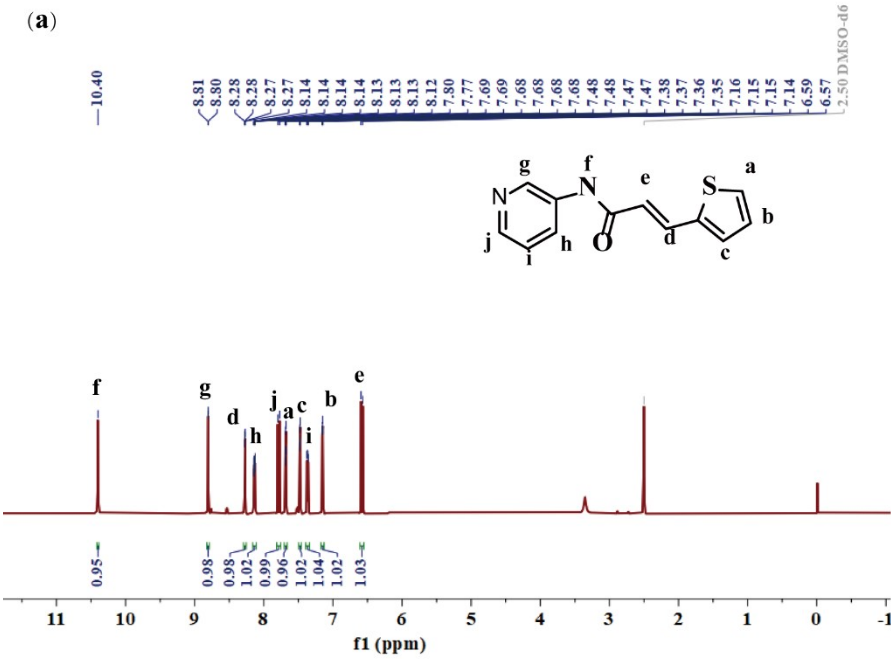

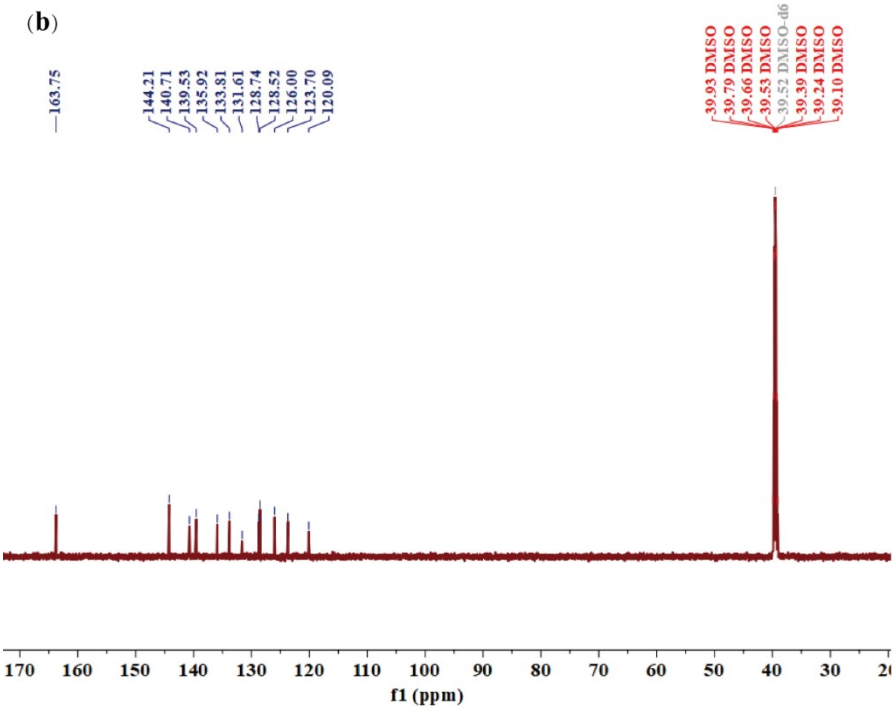

Fig. S6 ^{1}H-NMR spectrum (a) and ^{13}C-NMR spectrum (b) of **3-ptpa** in DMSO-d_6.

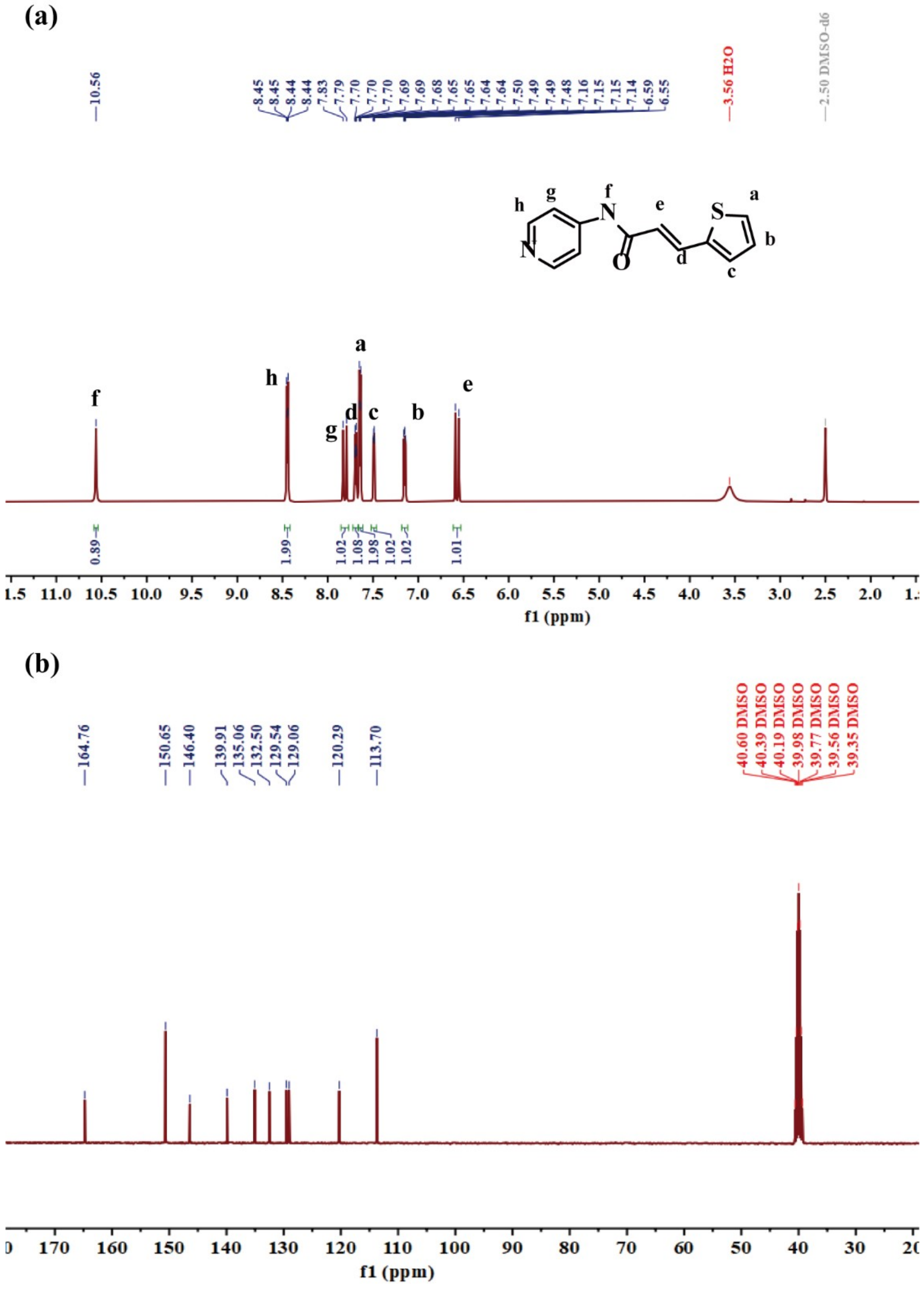

Fig. S7 ^{1}H-NMR spectrum (a) and ^{13}C-NMR spectrum (b) of **4-ptpa** in DMSO-d_6.

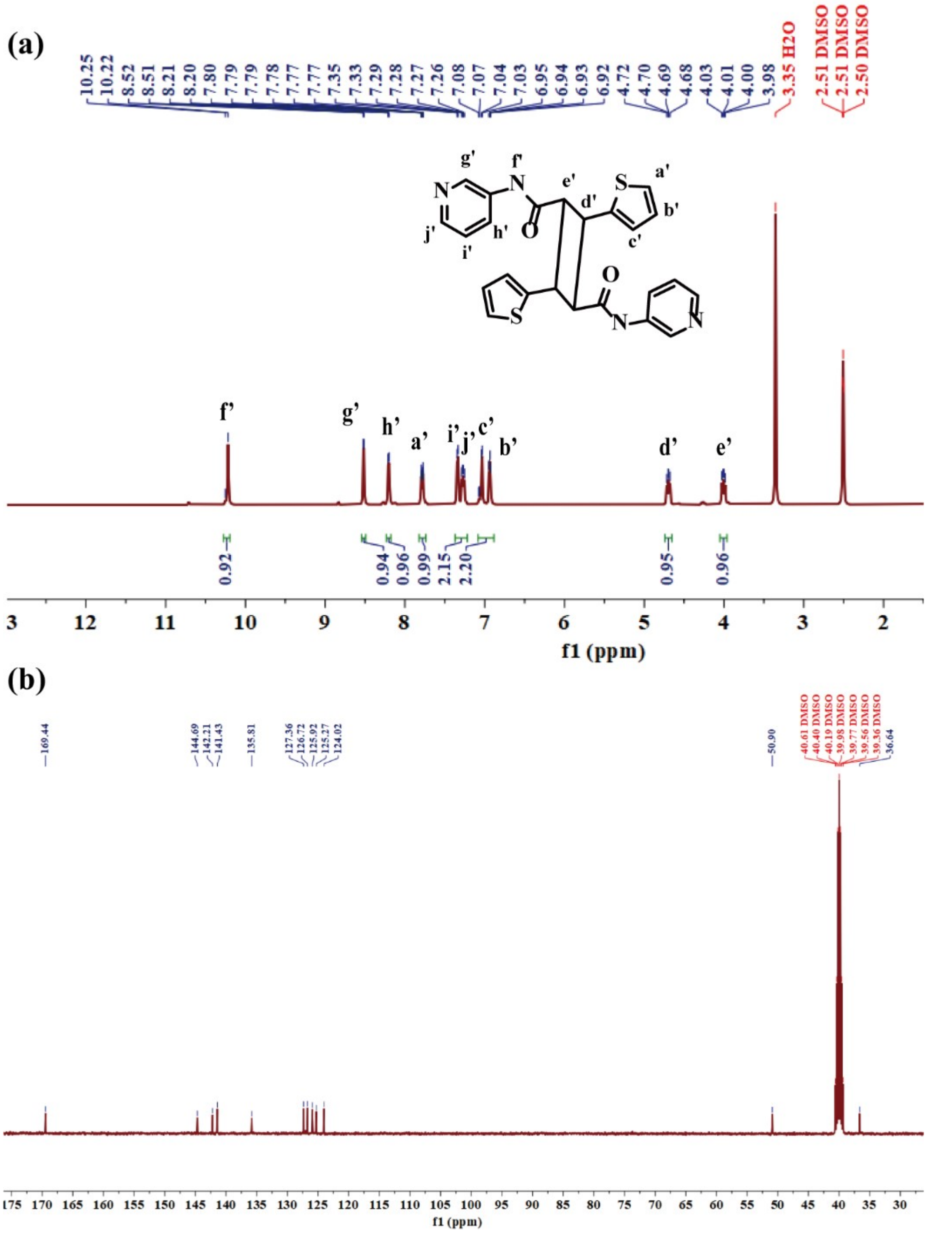

Fig. S8 ^{1}H-NMR spectrum (a) and ^{13}C-NMR spectrum (b) of $\mathbf{L_1}$ in DMSO-d_6.

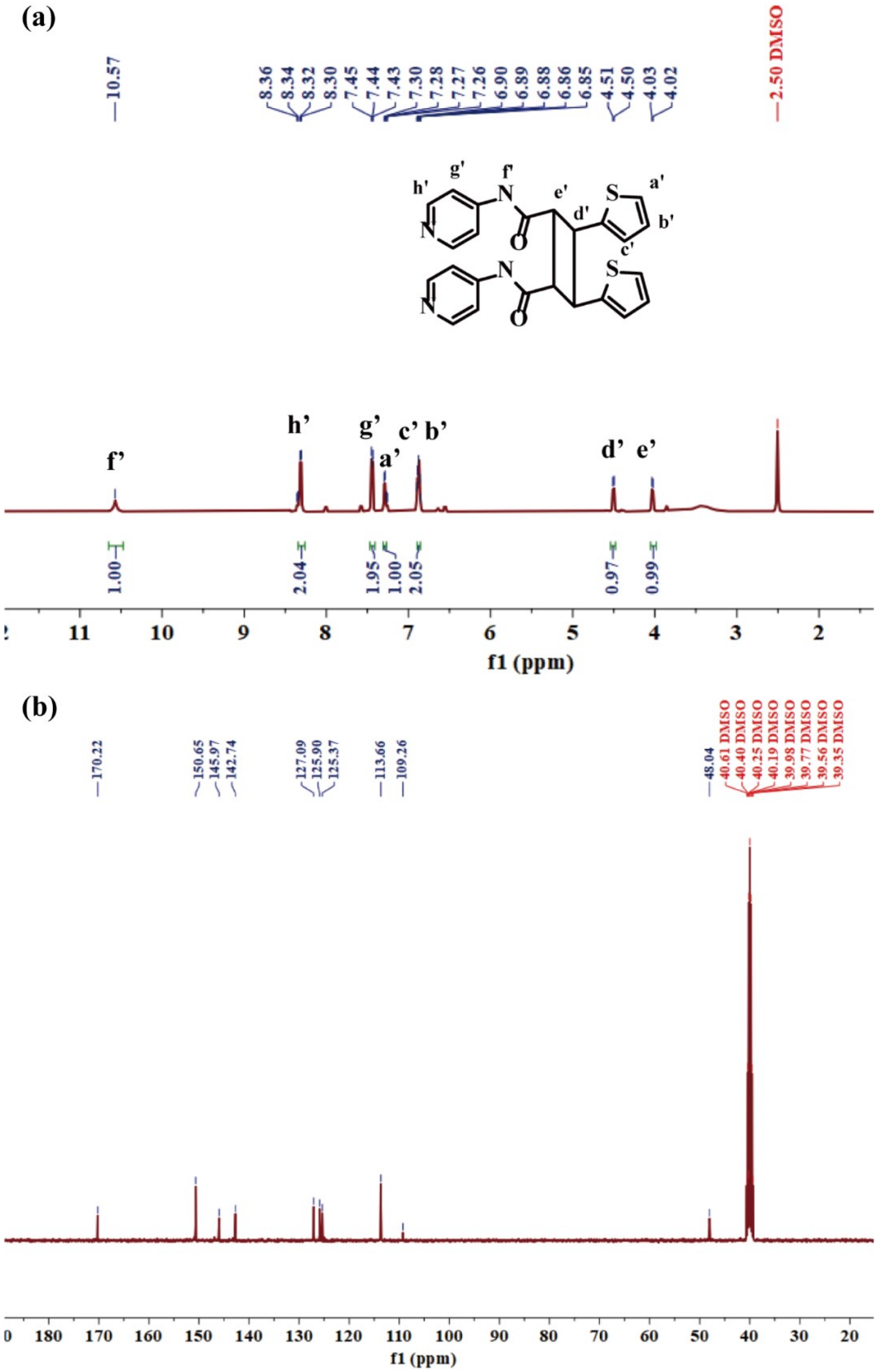

Fig. S9 ^{1}H-NMR spectrum (a) and ^{13}C-NMR spectrum (b) of $\mathbf{L_3}$ in DMSO-d_6.

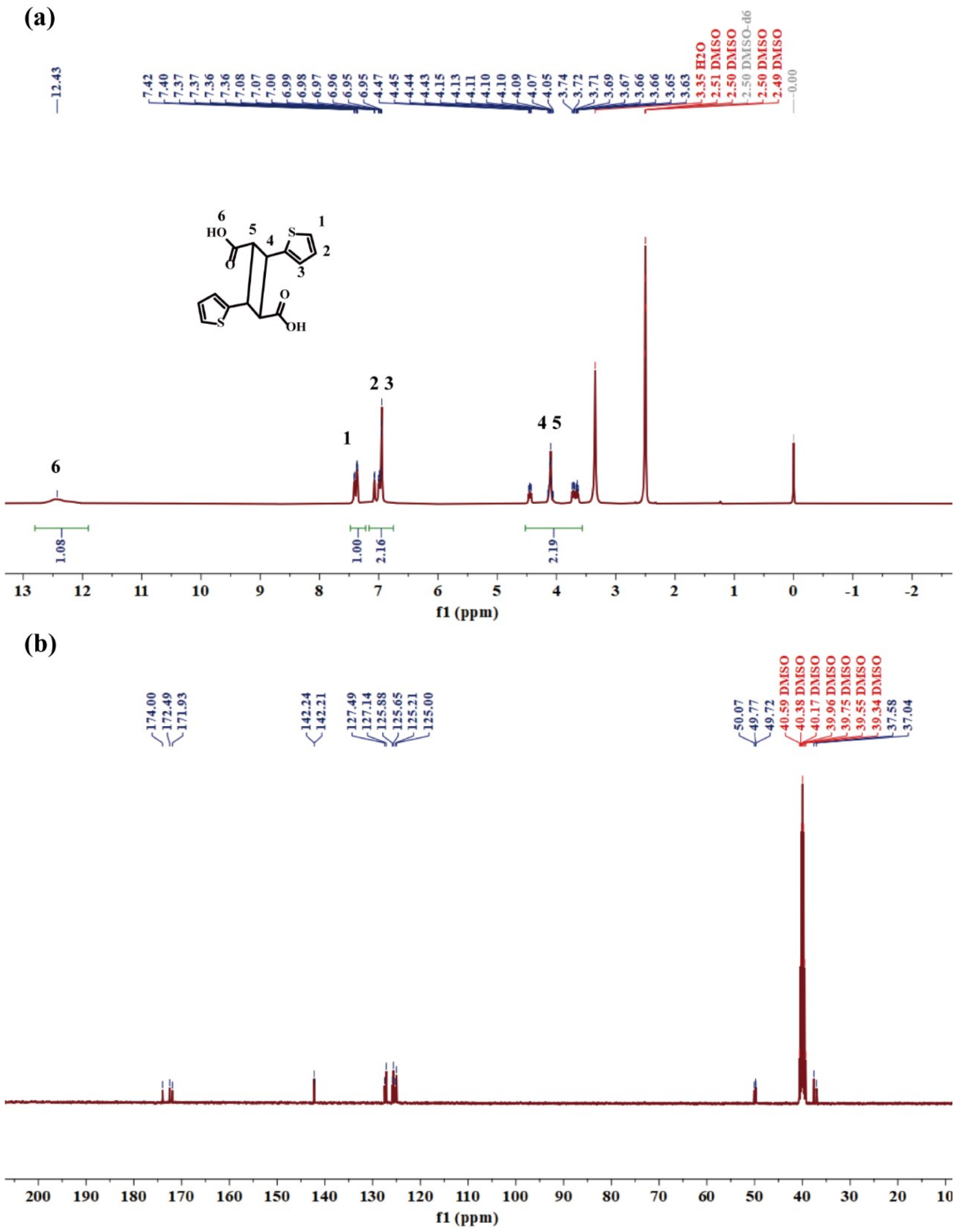

Fig. S10 ^{1}H-NMR spectrum (a) and ^{13}C-NMR spectrum (b) of $\mathbf{L_2}$ in DMSO-d_6.

(a)

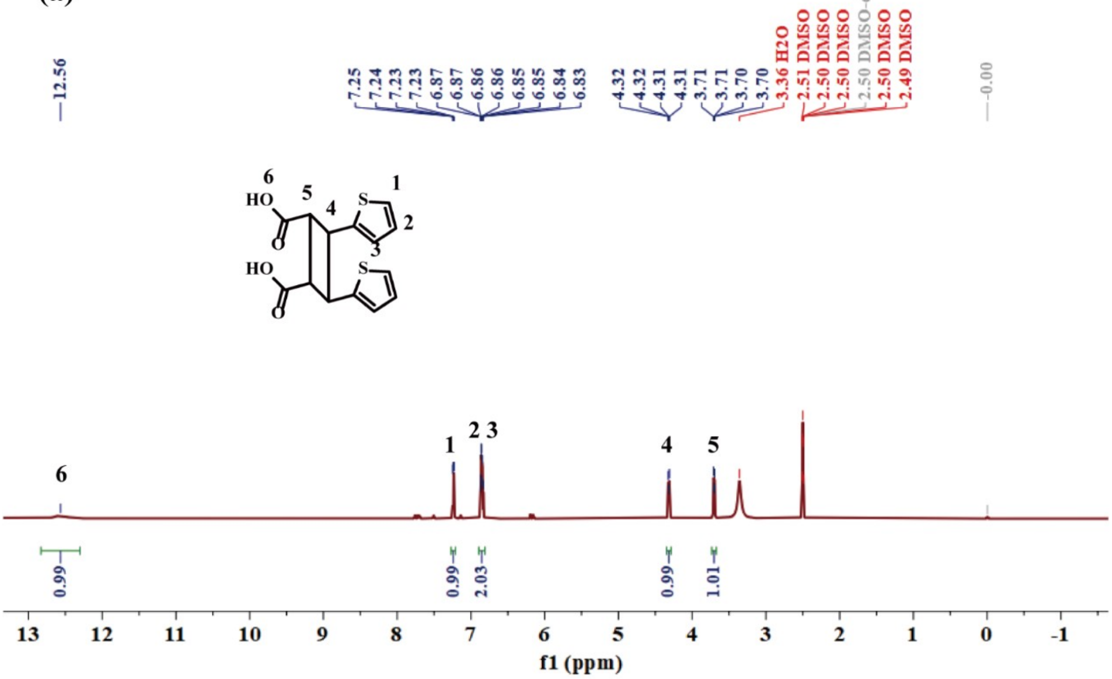

(b)

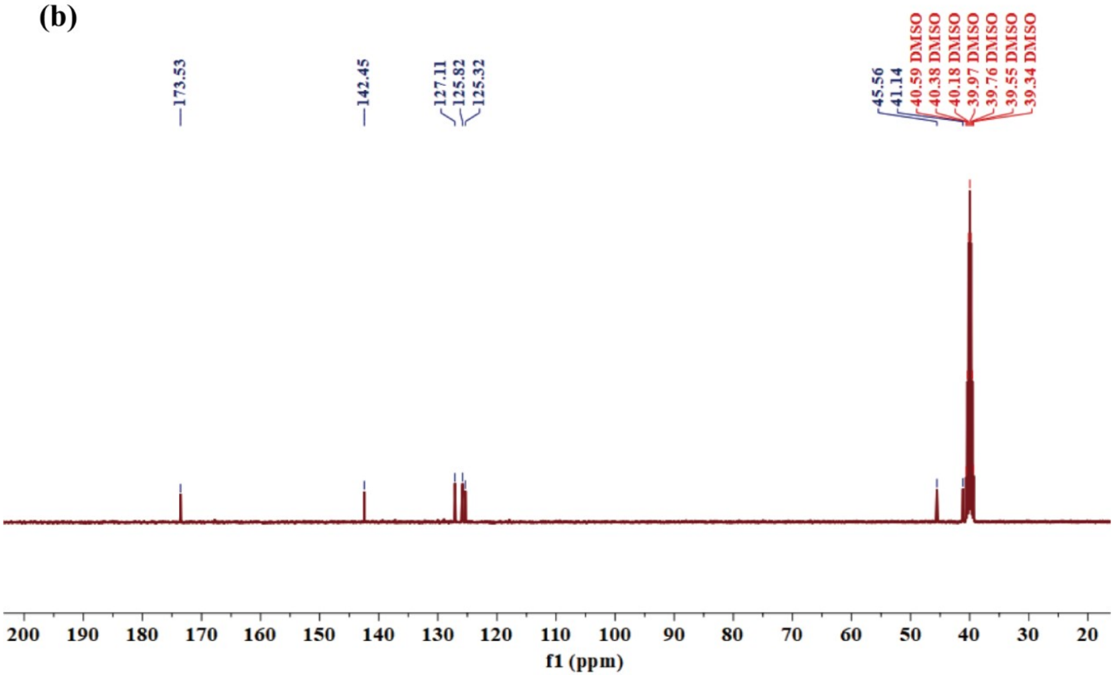

Fig. S11 ^{1}H-NMR spectrum (a) and ^{13}C-NMR spectrum (b) of **L_4** in DMSO-d_6.

Mass spectra

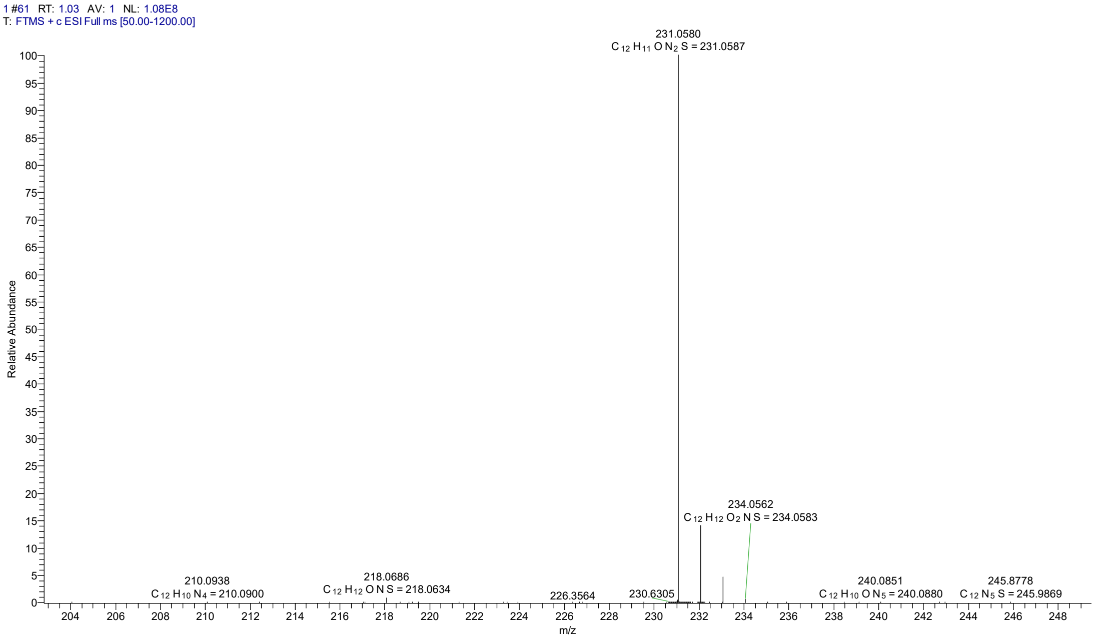

Fig. S12 Positive-ion ESI mass spectrum of **3-ptpa** in MeOH.

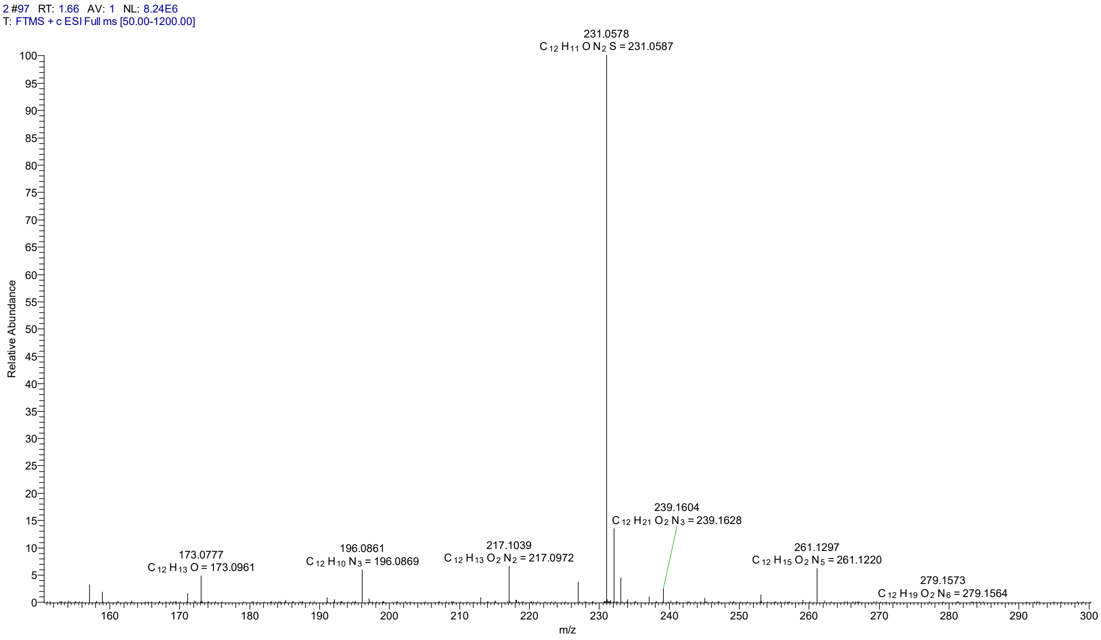

Fig. S13 Positive-ion ESI mass spectrum of **4-ptpa** in MeOH.

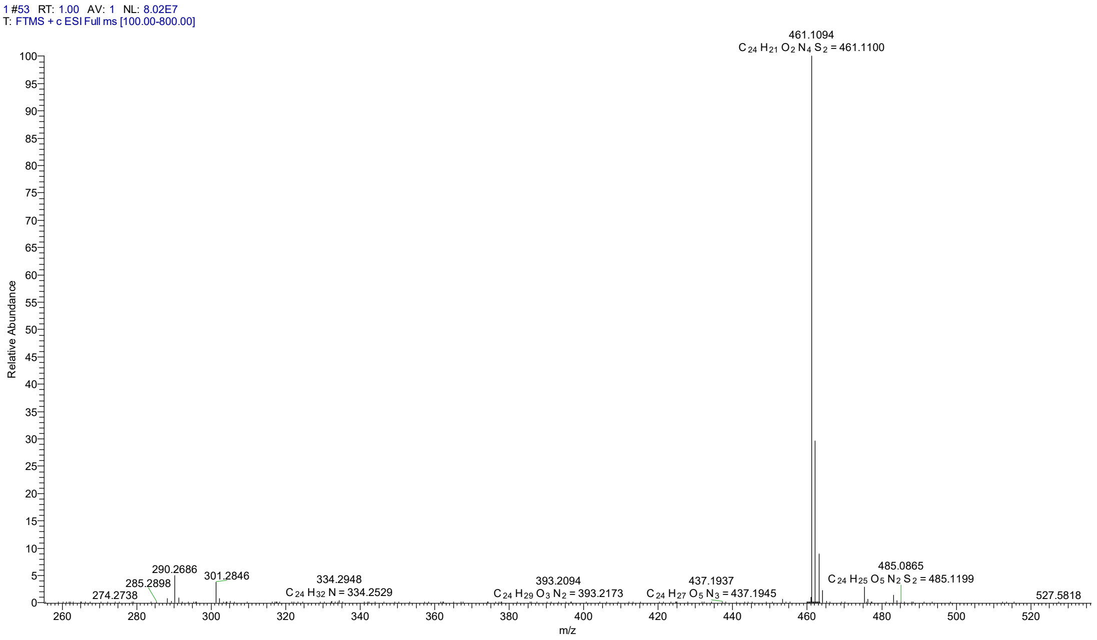

Fig. S14 Positive-ion ESI mass spectrum of $\mathbf{L_1}$ in MeOH.

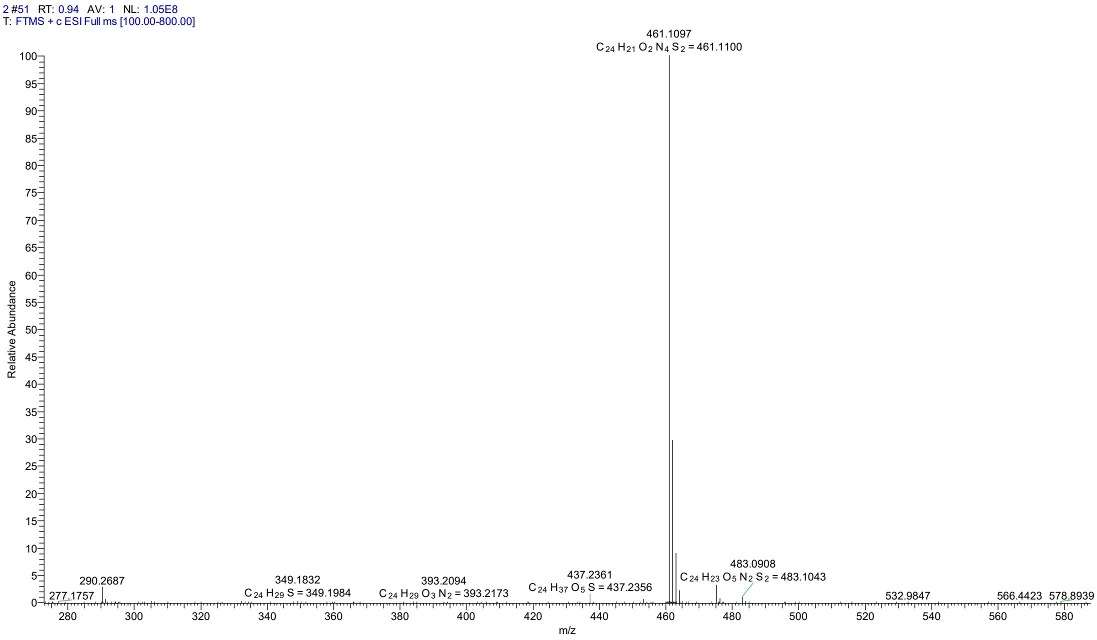

Fig. S15 Positive-ion ESI mass spectrum of $\mathbf{L_2}$ in MeOH.

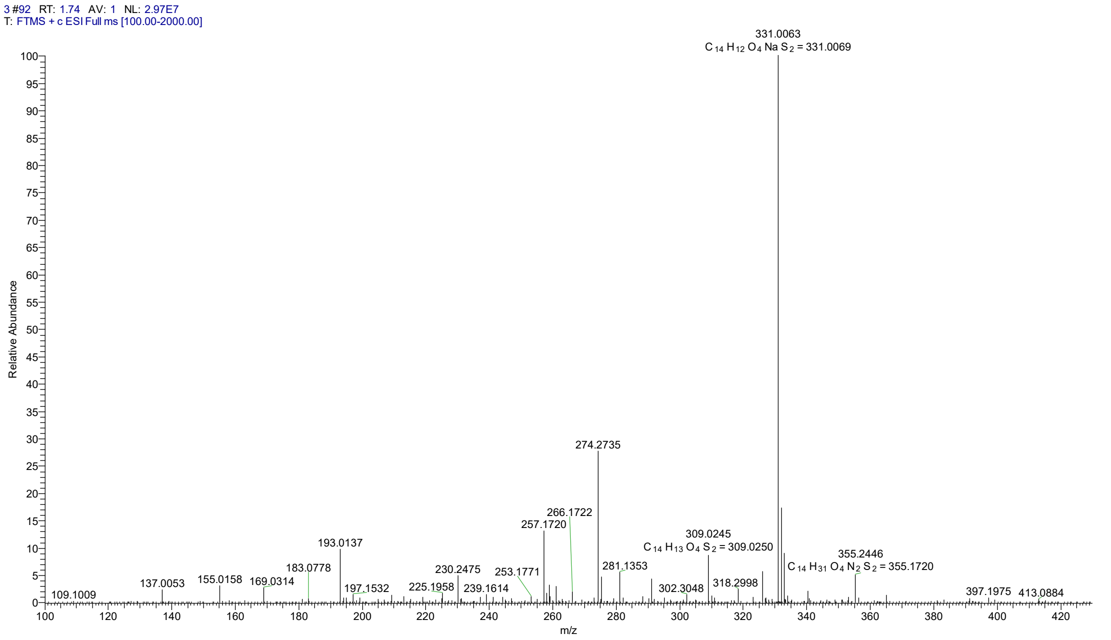

Fig. S16 Positive-ion ESI mass spectrum of $\mathbf{L_3}$ in MeOH.

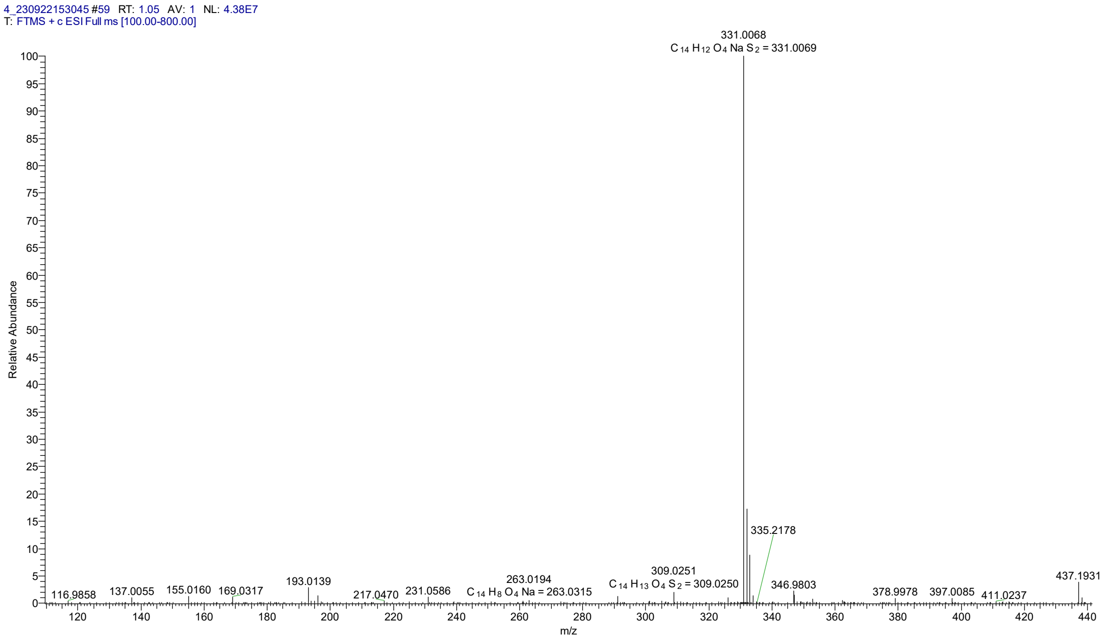

Fig. S17 Positive-ion ESI mass spectrum of $\mathbf{L_4}$ in MeOH.

IR spectra

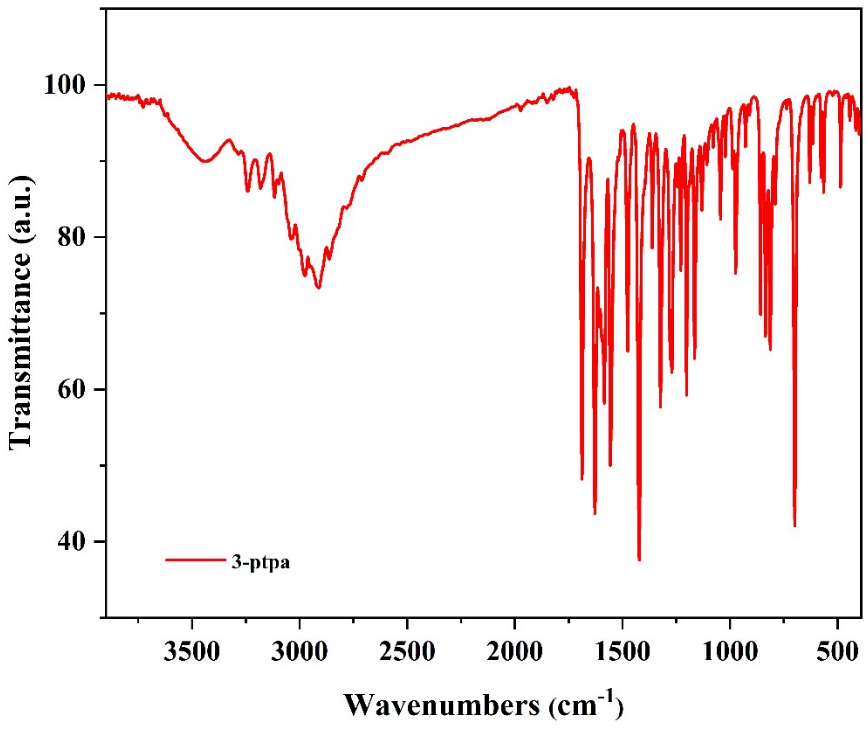

Fig. S18 IR spectra of **3-ptpa**.

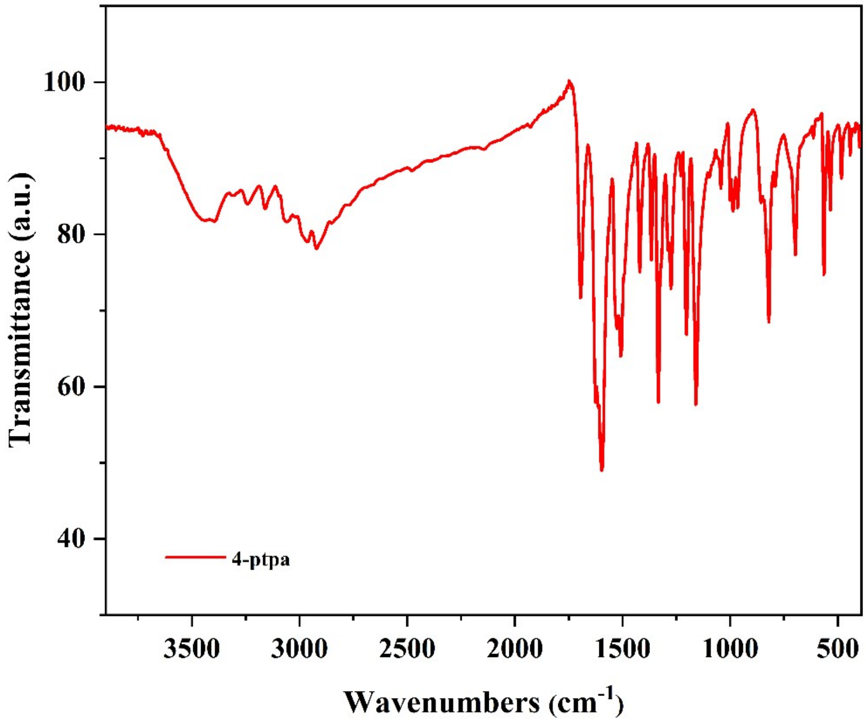

Fig. S19 IR spectra of **4-ptpa**.

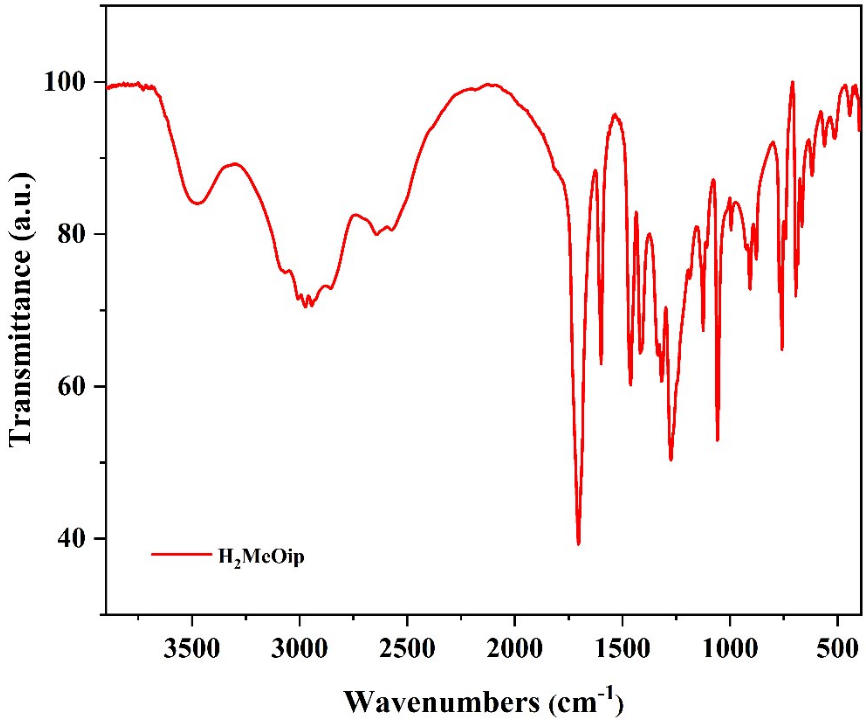

Fig. S20 IR spectra of H_2MeOip.

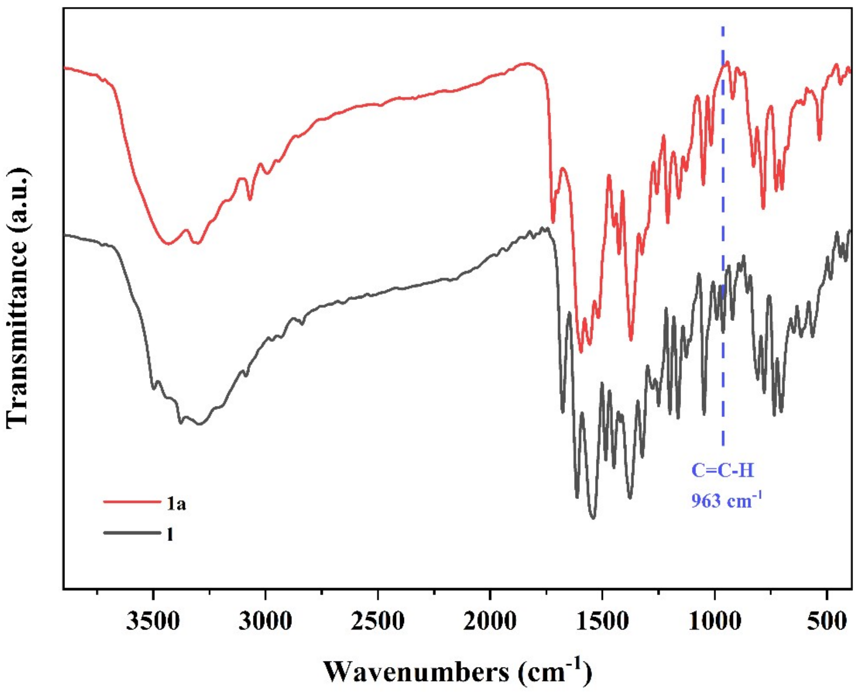

Fig. S21 IR spectra of **1** and **1a**.

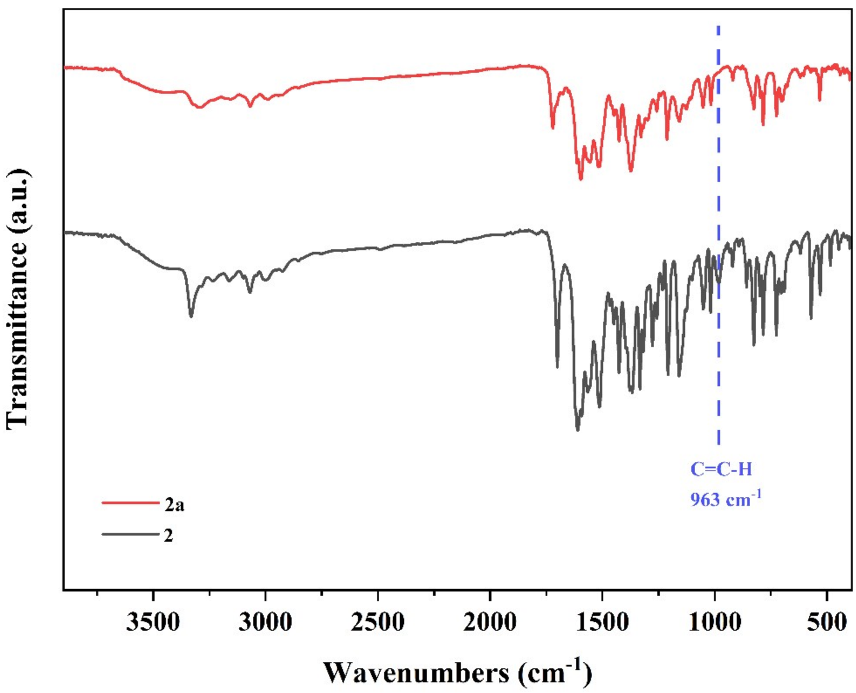

Fig. S22 IR spectra of **2** and **2a**.

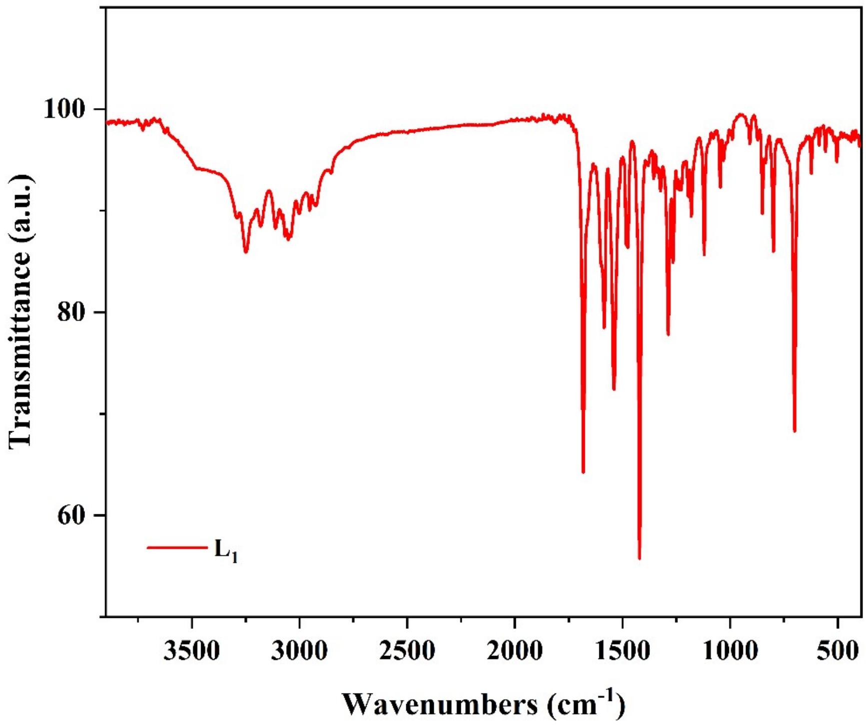

Fig. S23 IR spectra of $\mathbf{L_1}$.

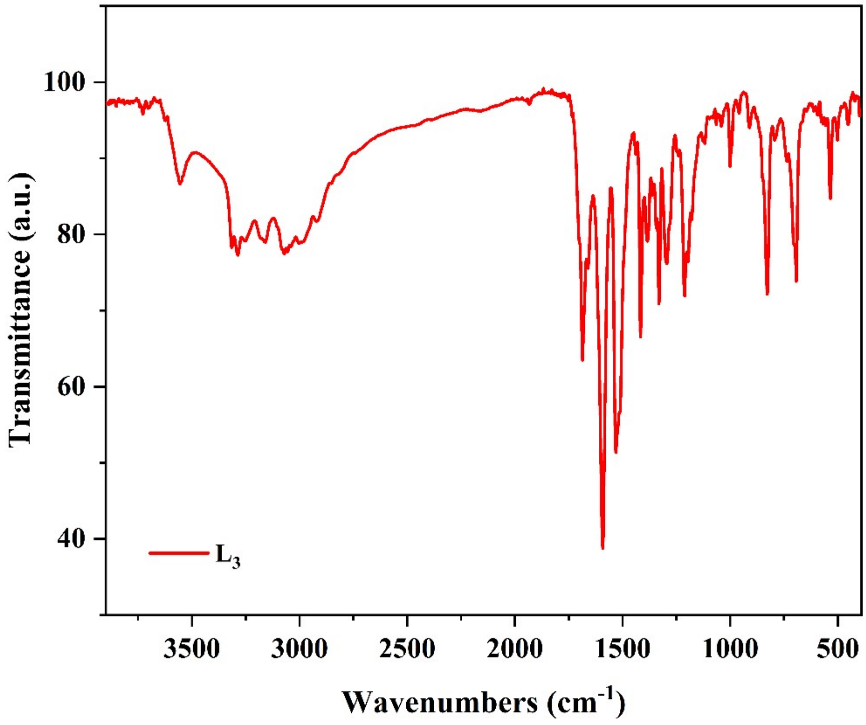

Fig. S24 IR spectra of **L_3**.

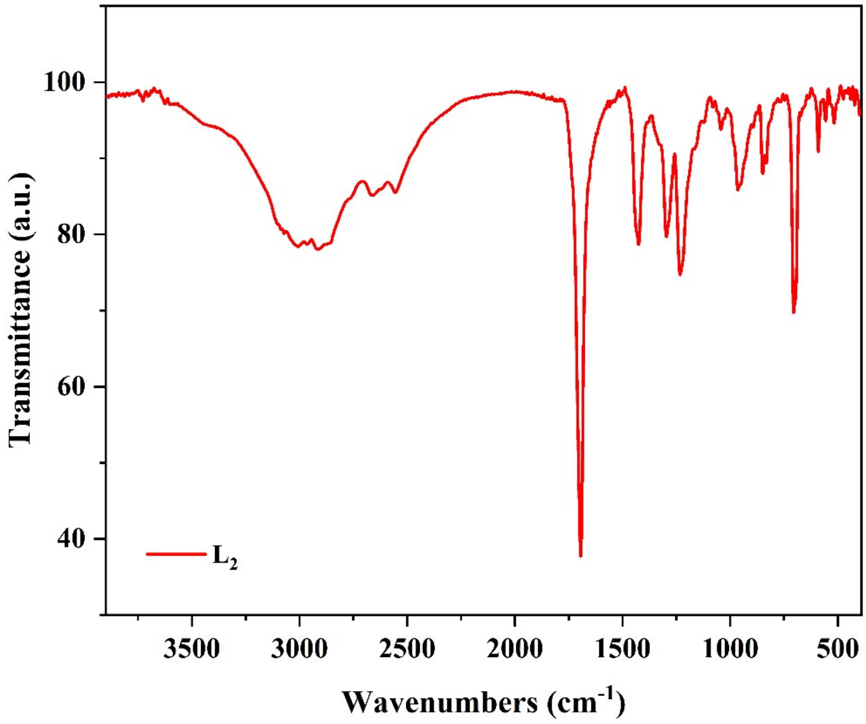

Fig. S25 IR spectra of $\mathbf{L_2}$.

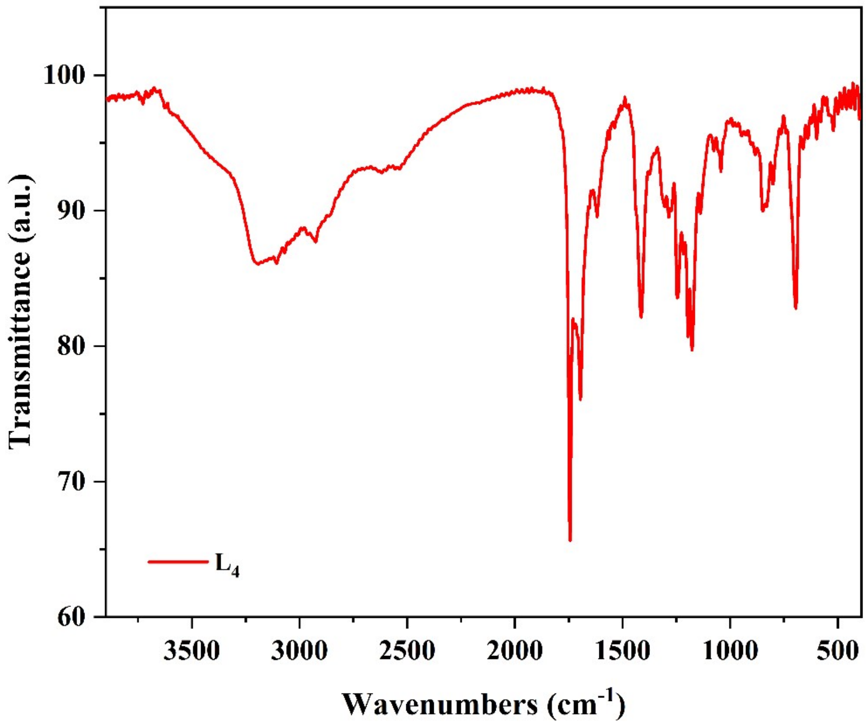

Fig. S26 IR spectra of $\mathbf{L_4}$.

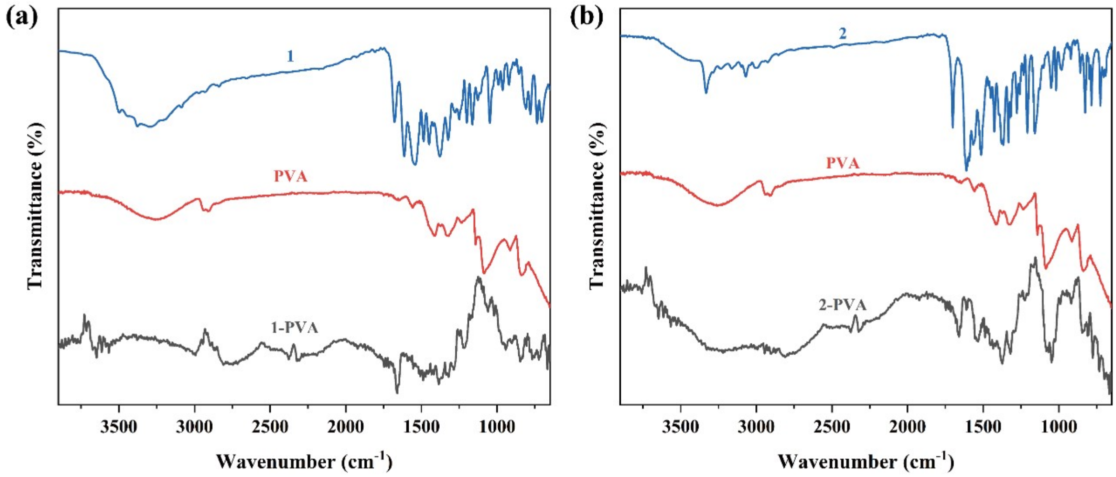

Fig. S27 IR spectra of **1-PVA** (a) and **2-PVA** (b).

Thermo gravimertic analysis (TGA)

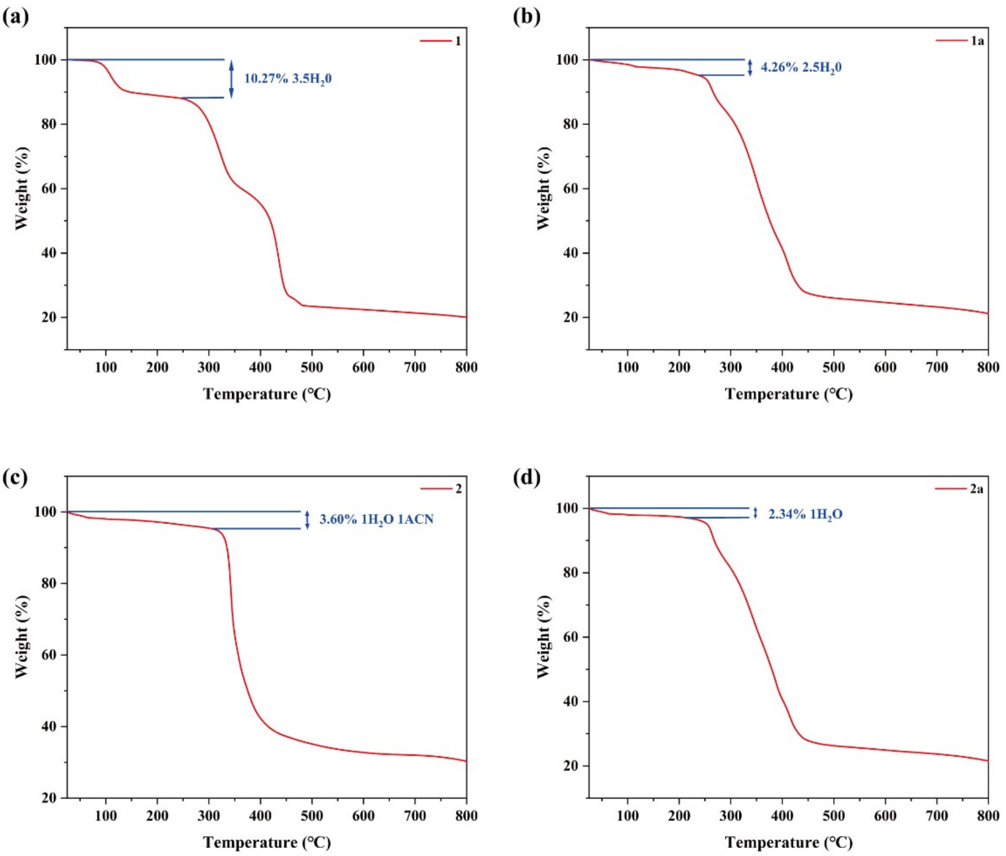

Fig. S28 TGA of **1**–**2a**.

The results of single crystal data show that there are four coordination water molecules in **1a**. This can be attributed to the loss of water molecules caused by the evaporation of a part of the water molecules under ultraviolet light. Therefore, the number of water molecules calculated by the TGA is smaller than the number of water molecules observed in the crystal data. We respected the experimental results and wrote the molecular formula according to the conclusions.

Solid–state UV–visible absorbance spectra

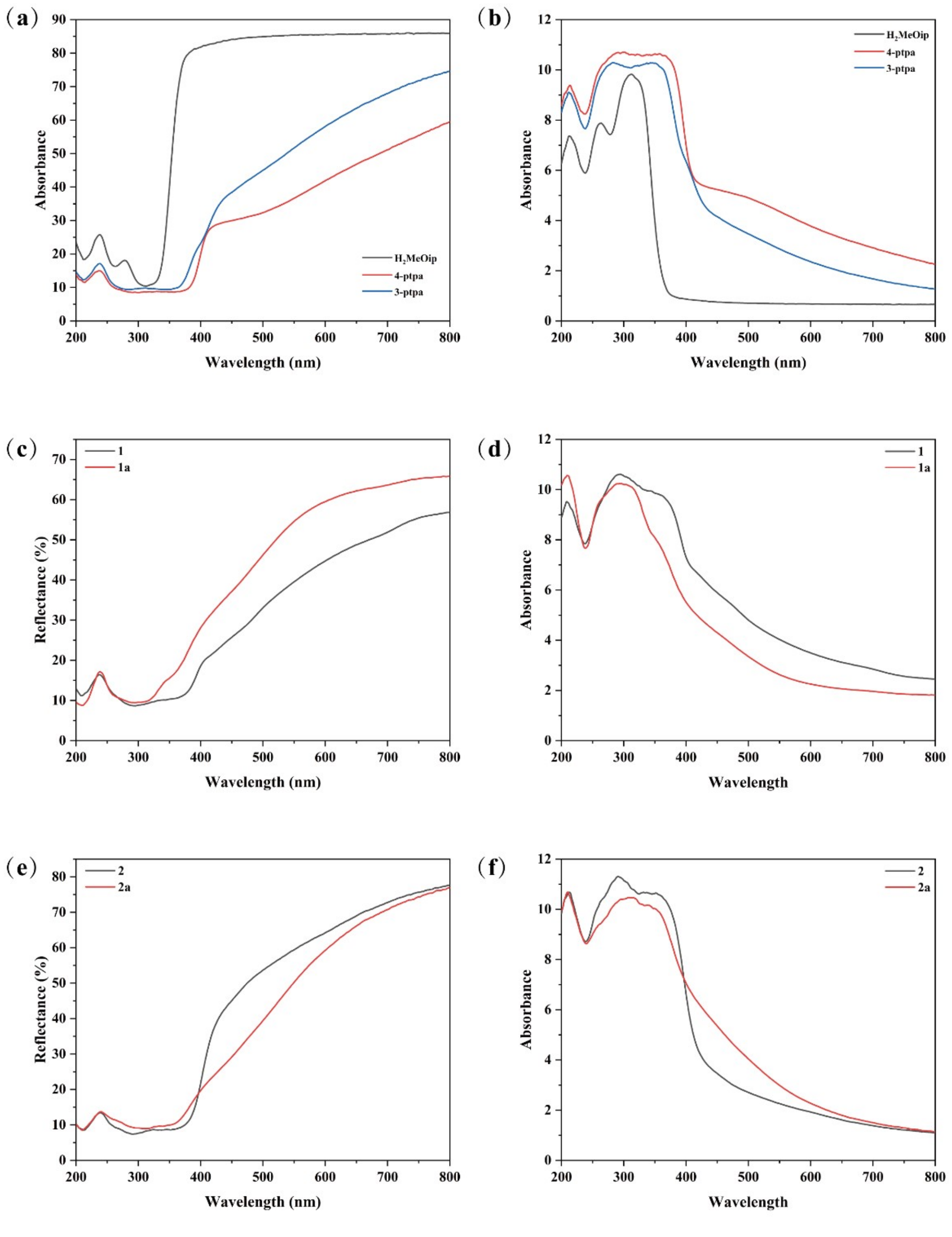

Fig. S29 Solid–state UV–visible absorbance.

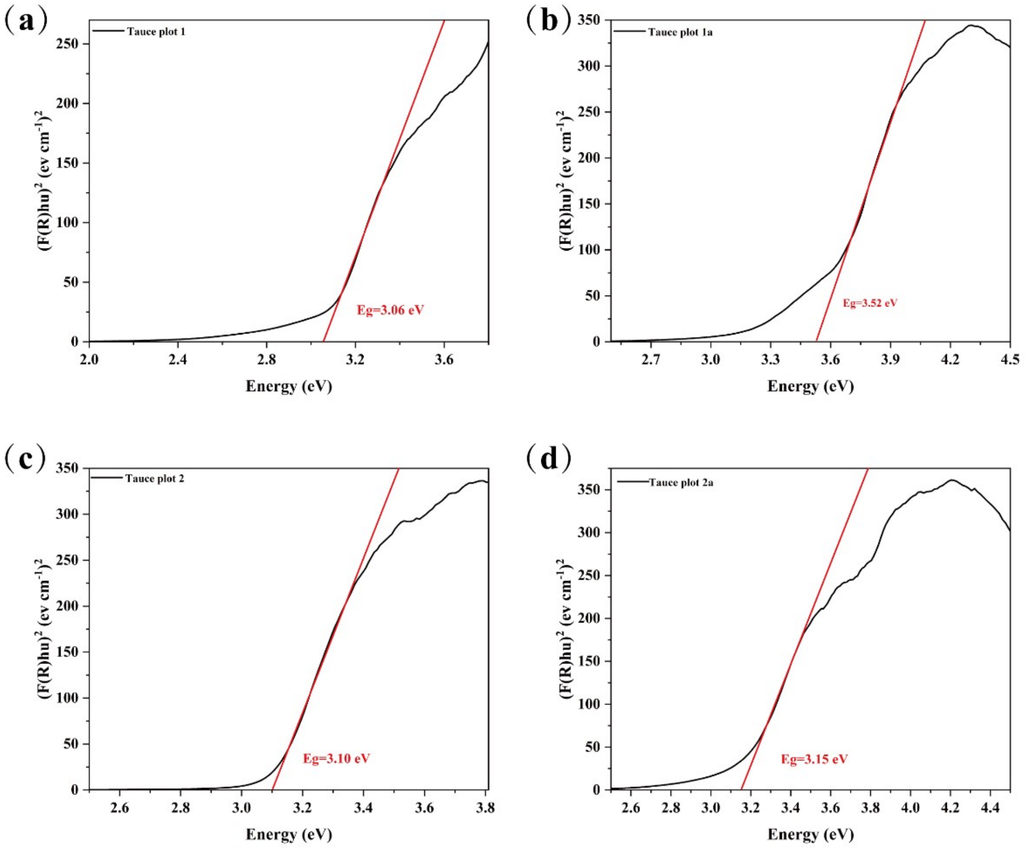

Fig. S30 the direct optical band gap.

Scanning electron microscope

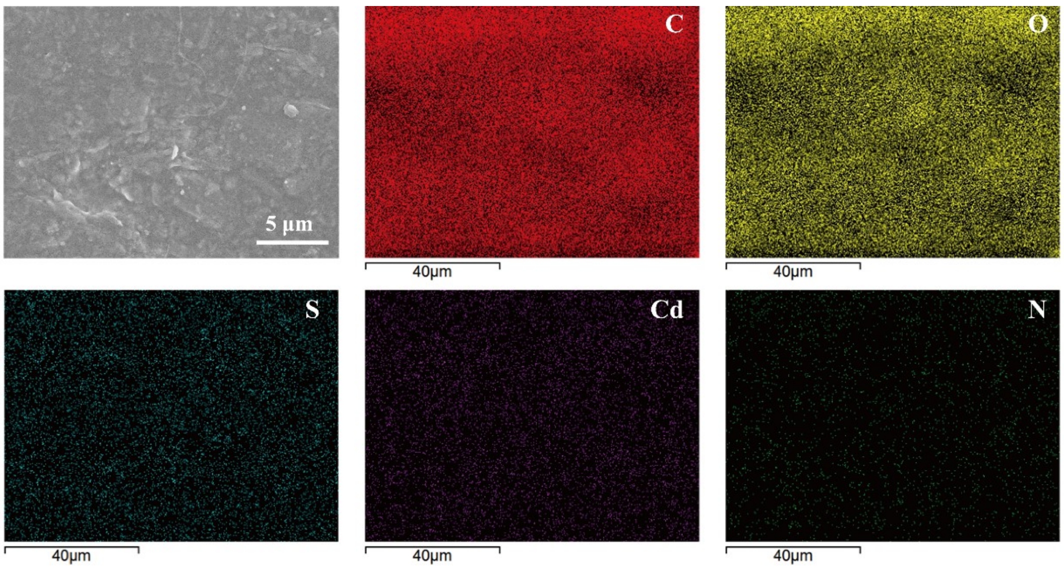

Fig. S31 SEM image and EDS mapping images of **1-PVA** membrane.

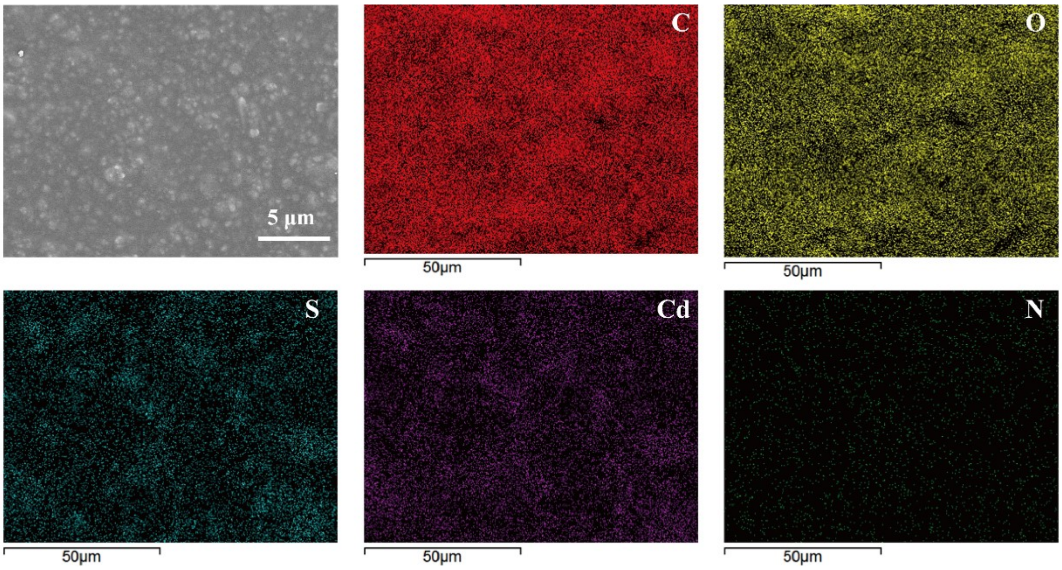

Fig. S32 SEM image and EDS mapping images of **2-PVA** membrane.

Photosalient Effects

Table S6 Single crystal data.

Date (RT)	1	1b	1c	2	2a
a (Å)	10.25	10.26	10.26	12.60	10.52
b (Å)	10.37	10.37	10.30	16.00	10.73
c (Å)	12.33	12.37	12.49	16.60	14.86
α (°)	93	93	93	88	93
β (°)	101	102	102	89	96
γ (°)	118	119	118	85	106
V ($Å^3$)	1106	1112	1121	3331	1594

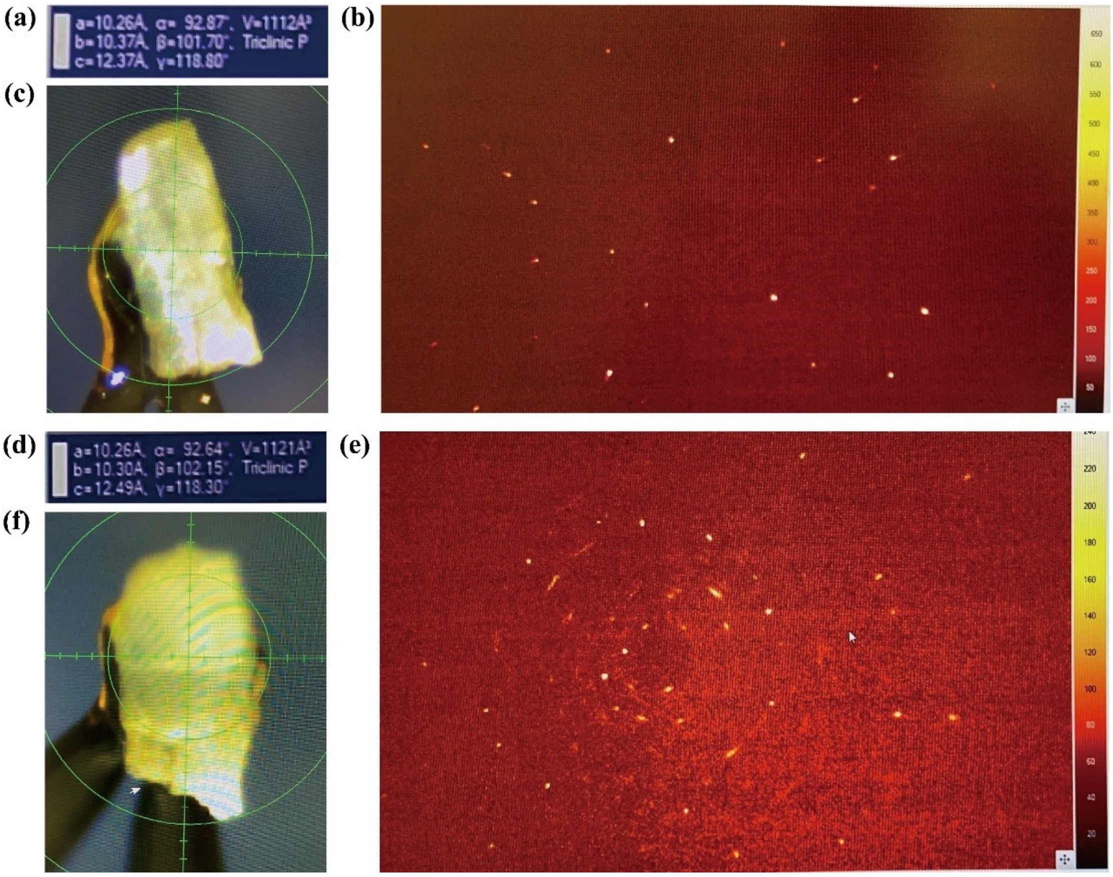

Fig. S33 X-ray diffraction analysis of crystals **1b** and **1c**. (a)(d) Cell parameters for crystals **1b** and **1c**. (b)(e) Diffraction images of crystals **1b** and **1c**. (c)(f) Crystals of **1b** and **1c**.

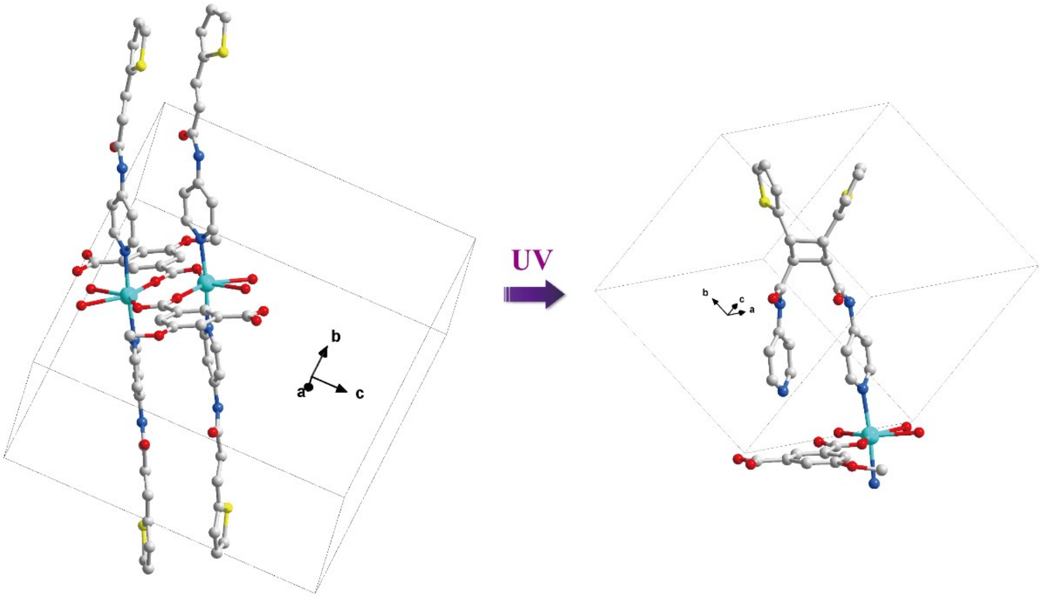

Fig. S34 Under UV light, the single crystal **2** becomes crystal **2a**, and the cell volume shrinks twice.

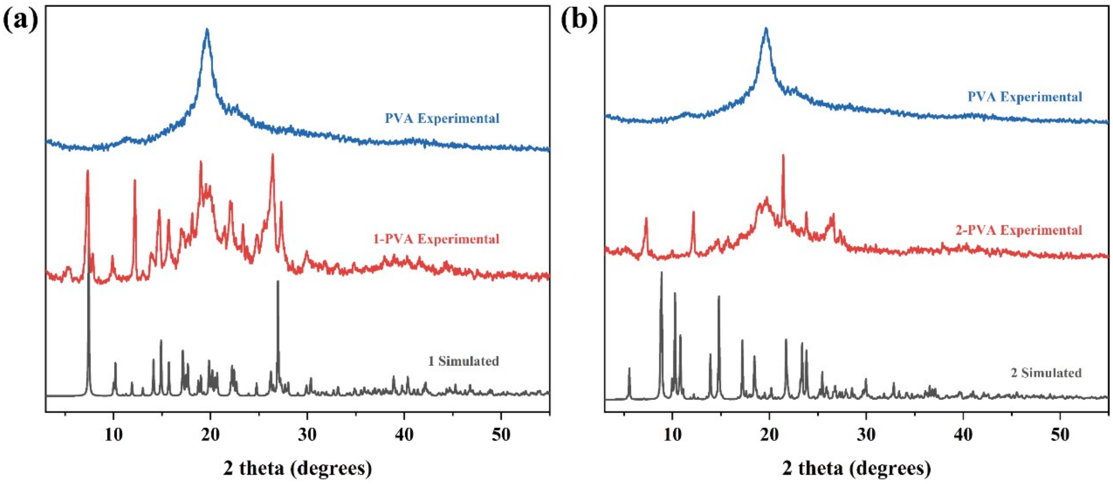

Fig. S35 PXRD profile of **1-PVA** membrane (a) and **2-PVA** membrane (b).

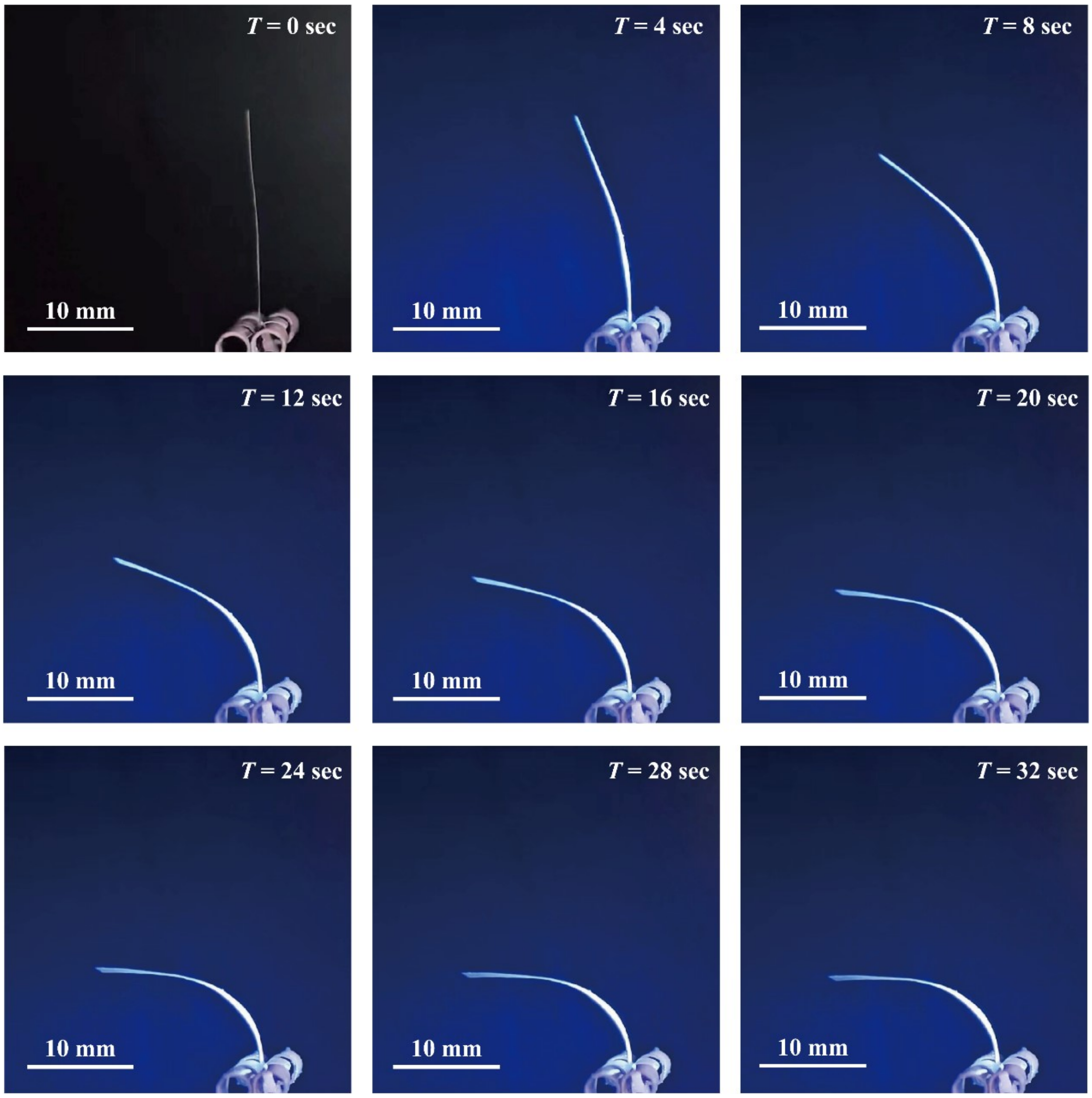

Fig. S36 Optical images of the composite membrane **1-PVA** upon exposure to 365 nm light.

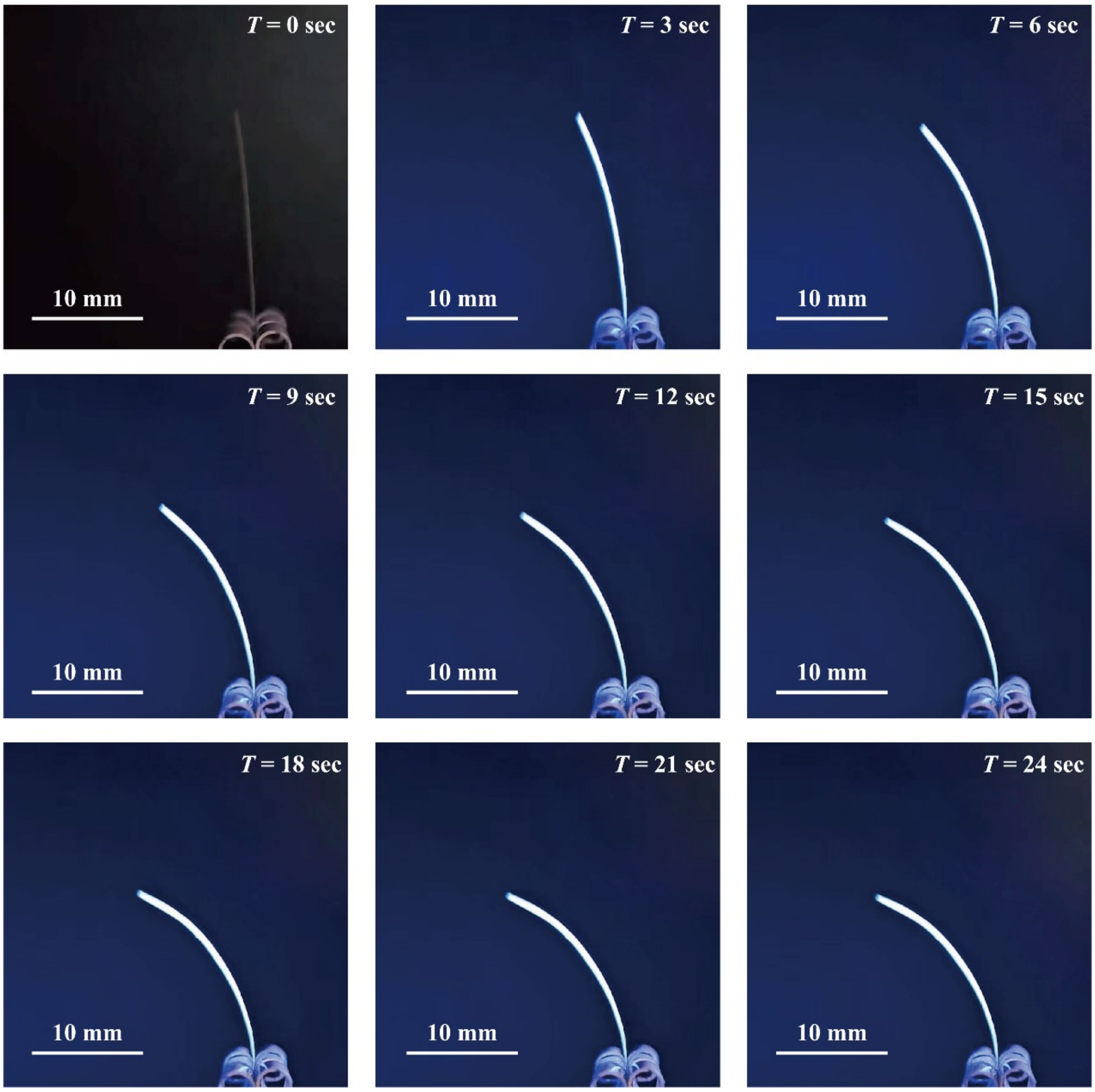

Fig. S37 Optical images of the composite membrane **2-PVA** upon exposure to 365 nm light.

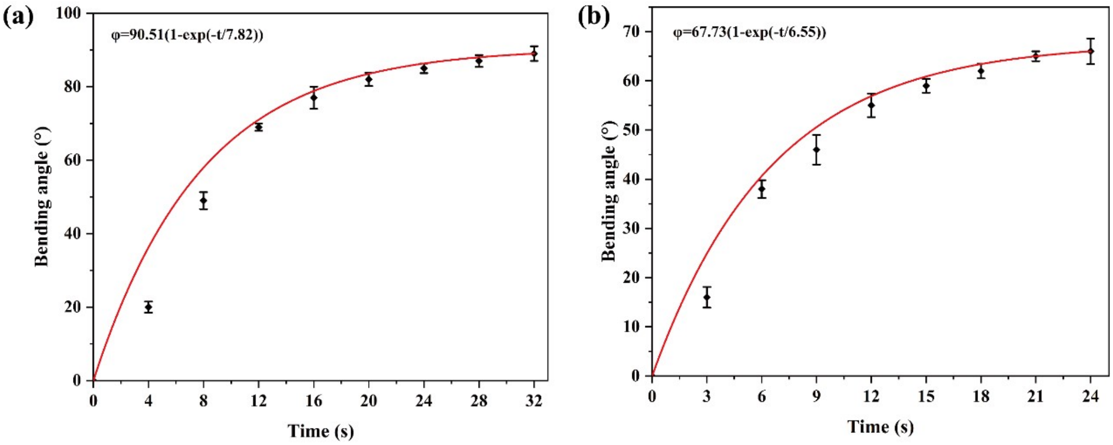

Fig. S38 Curves of **1-pva** (a) and **2-pva** (b) bending against exposure time.

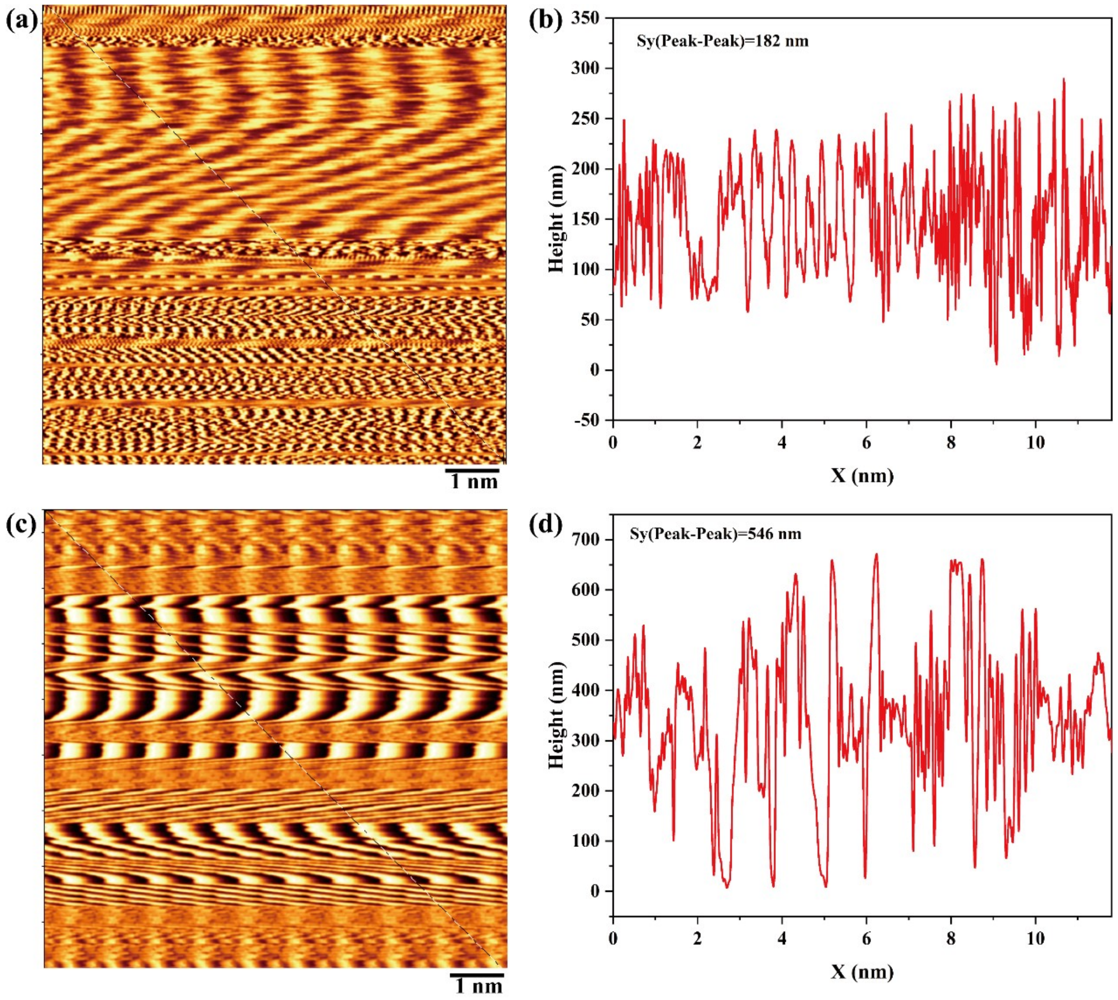

Fig. S39 AFM images and the corresponding height profiles of a section of a line in **1-PVA** (a, b) and irradiated **1-PVA** membrane (c, d).

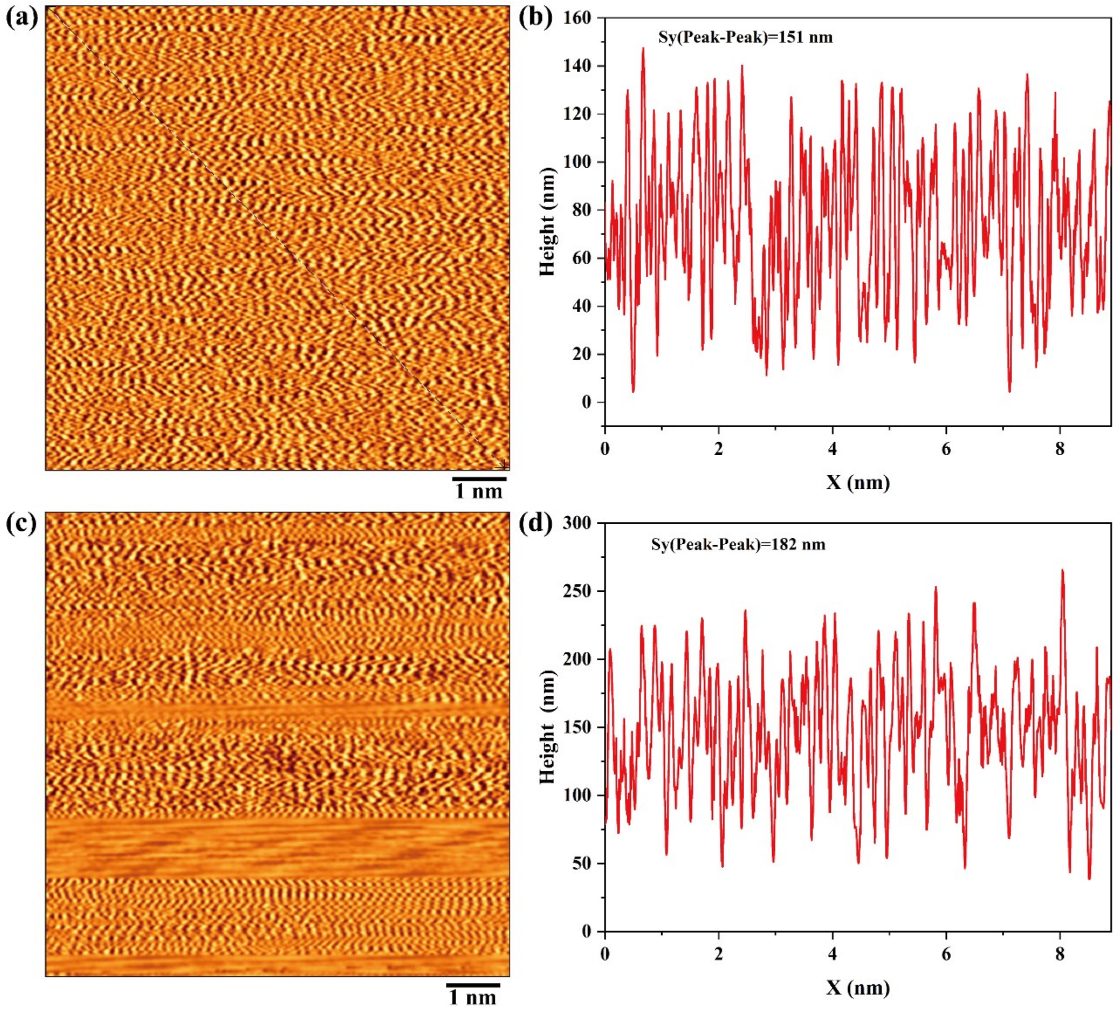

Fig. S40 AFM images and the corresponding height profiles of a section of a line in **2-PVA** (a, b) and irradiated **2-PVA** membrane (c, d).

References

1. R. Blessing, An empirical correction for absorption anisotropy, *Acta Crystallographica Section A*, 1995, **51**, 33-38.
2. G. Sheldrick, Crystal structure refinement with SHELXL, *Acta Crystallographica Section C*, 2015, **71**, 3-8.
3. O. V. Dolomanov, L. J. Bourhis, R. J. Gildea, J. A. K. Howard and H. Puschmann, OLEX2: a complete structure solution, refinement and analysis program, *Journal of Applied Crystallography*, 2009, **42**, 339-341.
4. R. W. Cheary and A. A. Coelho, A fundamental parameters approach to X-ray line-profile fitting, J. Appl. Crystallogr., 1992, 25, 109-121.
5. Bruker, TOPAS, Bruker AXS Inc., Karlsruhe, Germany, 7 edn., 2017.
